# Implementation strategies in emergency management of children: A scoping review

**DOI:** 10.1371/journal.pone.0248826

**Published:** 2021-03-24

**Authors:** Alex Aregbesola, Ahmed M. Abou-Setta, George N. Okoli, Maya M. Jeyaraman, Otto Lam, Viraj Kasireddy, Leslie Copstein, Nicole Askin, Kathryn M. Sibley, Terry P. Klassen

**Affiliations:** 1 The Children’s Hospital Research Institute of Manitoba, Winnipeg, Manitoba, Canada; 2 Department of Pediatrics and Child Health, Max Rady College of Medicine, Rady Faculty of Health Sciences, University of Manitoba, Winnipeg, Manitoba, Canada; 3 George & Fay Yee Centre for Healthcare Innovation, Max Rady College of Medicine, Rady Faculty of Health Sciences, University of Manitoba, Winnipeg, Manitoba, Canada; 4 Department of Community Health Sciences, Max Rady College of Medicine, Rady Faculty of Health Sciences, University of Manitoba, Winnipeg, Manitoba, Canada; 5 Neil John Maclean Health Sciences Library, University of Manitoba Libraries, University of Manitoba, Winnipeg, Manitoba, Canada; Zagazig University, EGYPT

## Abstract

**Background:**

Implementation strategies are vital for the uptake of evidence to improve health, healthcare delivery, and decision-making. Medical or mental emergencies may be life-threatening, especially in children, due to their unique physiological needs when presenting in the emergency departments (EDs). Thus, practice change in EDs attending to children requires evidence-informed considerations regarding the best approaches to implementing research evidence. We aimed to identify and map the characteristics of implementation strategies used in the emergency management of children.

**Methods:**

We conducted a scoping review using Arksey and O’Malley’s framework. We searched four databases [Medline (Ovid), Embase (Ovid), Cochrane Central (Wiley) and CINAHL (Ebsco)] from inception to May 2019, for implementation studies in children (≤21 years) in emergency settings. Two pairs of reviewers independently selected studies for inclusion and extracted the data. We performed a descriptive analysis of the included studies.

**Results:**

We included 87 studies from a total of 9,607 retrieved citations. Most of the studies were before and after study design (n = 68, 61%) conducted in North America (n = 63, 70%); less than one-tenth of the included studies (n = 7, 8%) were randomized controlled trials (RCTs). About one-third of the included studies used a single strategy to improve the uptake of research evidence. Dissemination strategies were more commonly utilized (n = 77, 89%) compared to other implementation strategies; process (n = 47, 54%), integration (n = 49, 56%), and capacity building and scale-up strategies (n = 13, 15%). Studies that adopted capacity building and scale-up as part of the strategies were most effective (100%) compared to dissemination (90%), process (88%) and integration (85%).

**Conclusions:**

Studies on implementation strategies in emergency management of children have mostly been non-randomized studies. This review suggests that ‘dissemination’ is the most common strategy used, and ‘capacity building and scale-up’ are the most effective strategies. Higher-quality evidence from randomized-controlled trials is needed to accurately assess the effectiveness of implementation strategies in emergency management of children.

## Introduction

While it would be ideal, not all hospitals have a separate pediatric emergency department (ED), and a significant number of children present at the general EDs [1, 2]. The requirements to manage pediatric emergencies differ from adults because of their unique needs in medication, equipment, staff, and pediatric-specific policies and protocols [3]. As new evidence is developed from well-designed research studies aimed at improving the health outcomes of children visiting EDs, it is critical to identify effective strategies to help implement new research findings to improve health outcomes [4].

In brief, implementation strategies are methods or techniques used to enhance the uptake and sustainability of research findings into routine practice [4]. They can be categorized into the following classes: (1) dissemination strategies: actions that target healthcare providers’ awareness, knowledge, attitudes, and intention to adopt an evidence-based intervention (EBI) [5], (2) process strategies: activities or processes related to quality improvement in planning, selecting and integrating EBI into practice [6], (3) integration strategies: activities or actions taken to address factors that positively or negatively influence optimal integration of specific EBI into practice [5], and (4) capacity building and scale-up strategies: strategies that target the general capacity of individuals to execute implementation process strategies [5]. These include training, technical assistance, tools, and opportunities for peer networking. An implementation strategy is described as being successful or effective when it leads to an increase in the uptake or utilization of guidelines, protocols or evidence into routine practice [7]. However, it remains unclear if study designs play a role in determining the effectiveness of implementation.

Accumulating implementation studies have continued to report on various implementation strategies used in the emergency management of children with inconclusive evidence on the effectiveness of the strategy used [8–12]. Apart from patient-measured outcomes, implementation studies are expected to also focus on healthcare professional and organizational behavior to accept or utilize evidence-based practices [7], but some studies neither investigated nor reported on it [13]. It is unclear what the characteristics of successful implementation strategies in EDs are. Thus, the aim of this scoping review is to identify and map the characteristics of implementation strategies in the emergency management of children.

## Methods

We used Arksey and O’Malley’s 5-stage framework to conduct our scoping review [14]. An a priori protocol of this study is available on the Open Science Framework platform (https://osf.io/h6jv2). Our review question was: What are the characteristics of successful implementation strategies used in the emergency management of children? We reported this review in accordance with the reporting guidance provided in the Preferred Reporting Items for Systematic Reviews and Meta-Analyses (PRISMA) Extension for Scoping Review Checklist (S1 Table) [15].

### Study eligibility criteria

This review included implementation studies conducted in EDs managing children (e.g., ≤21 years) [16]. Our intervention of interest was the use of any of the implementation strategies described earlier [5, 6]. We focused on controlled studies, defined in this case as studies that applied at least a guideline, protocol or a specific treatment plan compared to before implementation or to another setting in which the implementation strategy was not applied. Citations were limited to peer-reviewed, full-text articles published in English. There was no limit on the date of publication.

### Search strategy

A medical librarian (N.A.) designed and executed a literature search strategy in MEDLINE (Ovid) from inception through May 2019 (S2 Table). The search strategy was adapted for other bibliographic databases: Embase (Ovid), Cochrane Central (Wiley), and Cinahl (Ebsco). All retrieved citations were imported into an Endnote X8 (Clarivate Analytics, Philadelphia, PA).

### Study selection

**Fig 1:**
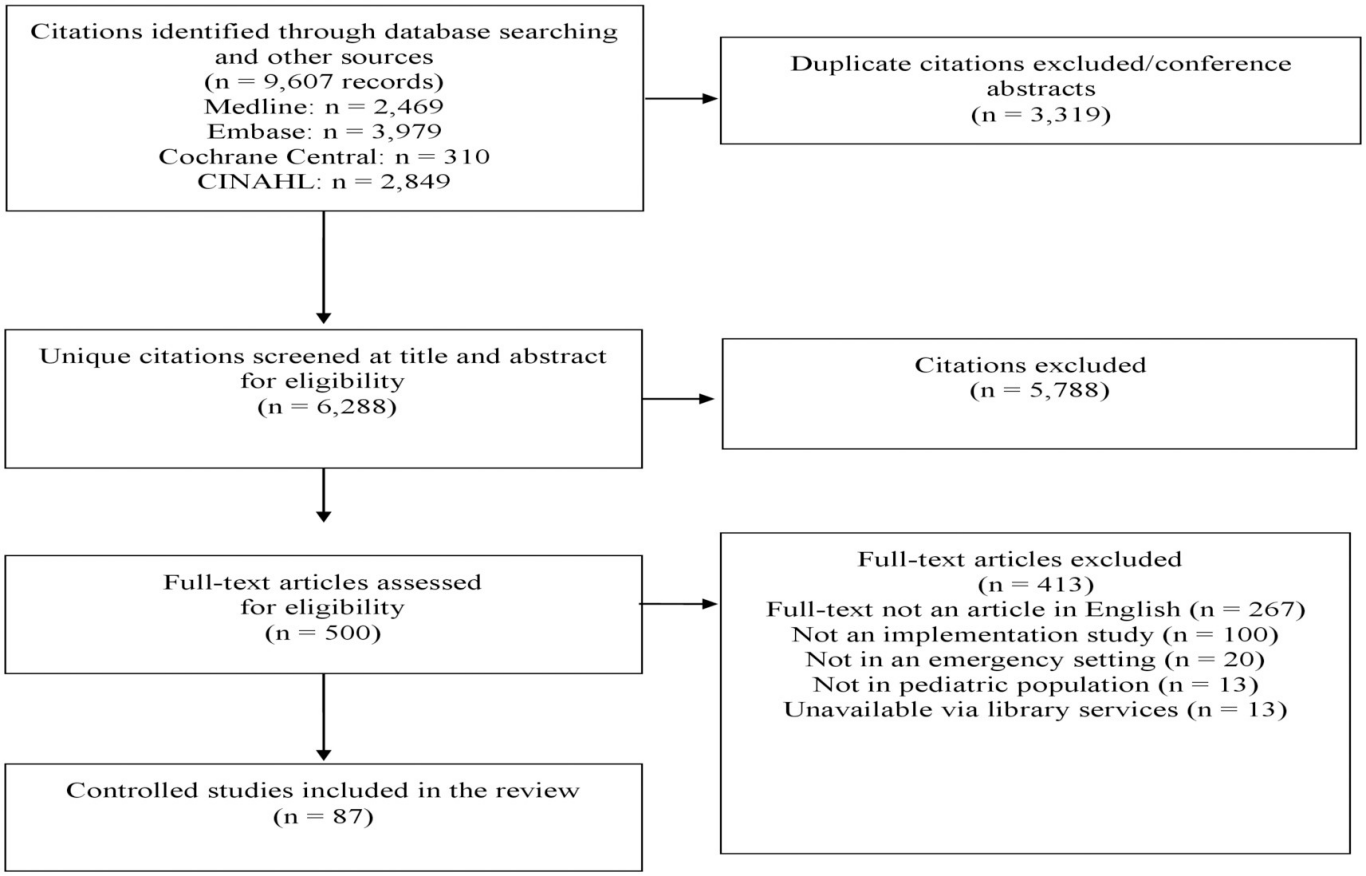
Summary of literature search and screening process.

Two pairs of reviewers (A.A., G.N.O., M.M.J., and O.L.) independently screened the identified citations for eligibility using a two-stage sifting approach to review the title, abstract, and full-text article. Disagreements between reviewers were observed on a few studies (<1%), which was resolved by a discussion between reviewers or by involving another reviewer (A.M.A.S.), on two occasions. We have reported the study selection process using the PRISMA flow diagram (Fig 1).

### Data extraction

Data were extracted using standardized pilot-tested forms, entered into MS Excel (Microsoft Corporation, Redmond, WA, USA) by one reviewer (A.A., G.N.O., M.M.J., or O.L.), and verified for accuracy and completeness by a second reviewer. Disagreements were resolved by discussion between reviewers or by involving another reviewer (A.M.A.S.), when necessary. We extracted data on the study details: first author, year of publication, study period, country, study design, study objective, area of study, and the intervention: use of any of the implementation strategies described earlier [5, 6], the effectiveness of the implementation strategies as it relates to healthcare provider-measured and patient-measured outcomes, and the number of strategies used.

### Risk of bias assessment

We did not appraise the risk of bias of the included studies, which is consistent with established scoping review methods [17].

### Data analysis

We performed screening and data management using MS Excel (Microsoft Corporation, Redmond, WA, USA). A descriptive analysis of different implementation strategies and the effectiveness was conducted and presented in tabular and narrative formats. We determined the effectiveness of the implementation strategy of the included studies based on the positive change reported in the outcomes of studies. We labelled the studies accordingly in cases where no change or negative change was observed in the outcomes. The effectiveness (%) of an implementation strategy was computed by dividing the number of studies that reported a positive effect (on the study outcome using the strategy) by the total number of studies that used the strategy [18, 19].

## Results

From 9,607 retrieved citations from four bibliographic databases, we included 87 studies [8–13, 20–100] (Fig 1). The detailed characteristics of the included studies are summarized in Table 1. Most of the included studies were from North America (n = 63, 70%) and Australia (n = 21, 11%). Before and after study design (n = 68, 61%) was the most common study design, and only (n = 7, 8%) were randomized controlled trials (RCTs) (S1 Fig).

**Table 1:** Characteristics of 87 included studies.

First author, year of publication	Study period	Region	Study design	Study area
McGrew, 2018 [8]	2011–2	North America	Before & after	Radiology
McLaughlin, 2018 [9]	2008–11	North America	Before & after	General PEM
Tavarez, 2017 [10]	NR	North America	Before & after	General PEM
Lee, 2018 [11]	2010–1	North America	Before & after	Anaphylaxis
Waddell, 2014 [12]	2002–5	North America	Before & after	General PEM
Johnson, 2018 [13]	NR	North America	Before & after	Asthma
Puffenbarger, 2019 [20]	2010–1	North America	Before & after	Radiology
Lukes, 2019 [21]	2013	North America	Cohort	Antibiotics
Carson, 2018 [22]	2011	North America	Before & after	Screening
Libetta, 1999 [23]	2009–11	Europe	Before & after	Radiology
Hendrickson, 2018 [24]	2010–2	North America	Before & after	Appendicitis
Norton, 2007 [25]	2008–9; 2010–1	North America	Before & after	Asthma
Dona, 2018 [26]	2008–9	Europe	Before & after	Antibiotics
Mohan, 2018 [27]	NR	North America	Non-RCT	Radiology
Jones, 2017 [28]	NR	Australia	Cohort	Triage/patient flow
Murray, 2017 [29]	2008–9	North America	Before & after	Fever
Geurts, 2017 [30]	2008–10	Europe	Randomized trial	Gastroenteritis
Ahmad, 2017 [31]	NR	North America	Before & after	Sepsis/infection
Gildenhuys, 2009 [32]	2005–9	Australia	Before & after	Asthma
Rutman, 2016 [33]	2005–9	North America	Interrupted time series	Asthma
Lin, 2016 [34]	2010	Asia	Before & after	Triage/patient flow
Shah, 2016 [35]	2007–9	North America	Before & after	Appendicitis
Dandoy, 2016 [36]	2009	North America	Time-series	Antibiotics
Cohen, 2016 [37]	2007	North America	Cohort	Antibiotics
Fallon, 2015 [38]	2002–7	North America	Before & after	Appendicitis
Jeong, 2015 [39]	2002; 2003–4	Asia	Before & after	Triage/patient flow
Dexheimer, 2014 [40]	1996–06	North America	Randomized trial	Asthma
Higginbotham, 2014 [41]	2009	North America	Before & after	Screening
Geurts, 2014 [42]	2007–8	Europe	Before & after	Sepsis/infection
Boutis, 2013 [43]	2008	North America	Interrupted time series	Radiology
Taylor, 2013 [44]	2006–7	Australia	Before & after	Pain management
Russell, 2013 [45]	2006	North America	Before & after	Triage/patient flow
Hack, 2013 [46]	2004–5	North America	Before & after	HIV
Wolff, 2012 [47]	2001–6	North America	Cohort	Jaundice
Doyle, 2012 [48]	2003–5	North America	Before & after	Triage/patient flow
Waseem, 2012 [49]	2004–5	North America	Before & after	Triage/patient flow
Hendrickson, 2012 [50]	2002–4	North America	Before & after	Transfusion
Crocker, 2012 [51]	2001–3	NR	Before & after	Pain management
Angoulvant, 2012 [52]	2000–2	Europe	Interrupted time series	Antibiotics
Larsen, 2011 [53]	1992–6	North America	Before & after	Sepsis/infection
Cruz, 2011 [54]	2000–1	North America	Before & after	Sepsis/infection
Iyer, 2011 [55]	1994–9	North America	Interrupted time series	Pain management
Fagbuyi, 2011 [56]	1997–8	North America	Before & after	Triage/patient flow
Fein, 2010 [57]	1997–9	North America	Before & after	Screening
Babl, 2010 [58]	1992–5	Australia	Before & after	Sedation
To, 2010 [59]	1985–91	North America	Before & after	Asthma
Trottier, 2010 [60]	2014–6	North America	Cohort	Migraine
Cruz, 2010 [61]	2009–11	North America	Cohort	Triage/patient flow
Burnette, 2009 [62]	2008	North America	Before & after	General PEM
Gauthier, 2009 [63]	2007–10	North America	Before & after	Sepsis/infection
Minniear, 2009 [64]	2005–8	North America	Before & after	HIV
Kozer, 2009 [65]	2002–4	Middle East	Cohort	Screening
Hayden, 2009 [66]	2003–4	Australia	Before & after	General PEM
Callegaro, 2009 [67]	1997–02	Europe	Before & after	Fever
Morrissey, 2009 [68]	1996–99	North America	Cohort	Pain management
Roukema, 2008 [69]	2010–1	Europe	Randomized trial	Fever
Doherty, 2007 [70]	2014–5	Australia	Before & after	Asthma
Boychuk, 2006 [71]	2014	North America	Before & after	Asthma
De Marco, 2005 [72]	2010–2	Europe	Case-control	Triage/patient flow
Buckmaster, 2005 [73]	NR	Australia/Europe	Before & after	Radiology
Buller-Close, 2003 [74]	2008	North America	Interrupted time series	Fever
Lee, 2003 [75]	2003–5	North America	Before & after	Radiology
Perlstein, 2002 [76]	NR	North America	Cohort	Gastroenteritis
Sharieff, 2001 [77]	2006–8	North America	Before & after	Antibiotics
Gazarian, 2001 [78]	1998–9	Australia	Before & after	Asthma
Schriger, 2000 [79]	1997–8; 2001–2	North America	Interrupted time series	General PEM
Lavelle, 1998 [80]	2005	North America	Cohort	Triage/patient flow
Rooholamini, 2017 [81]	2010–1	North America	Before & after	General PEM
Hall, 2013 [82]	2013	North America	Before & after	General PEM
Zeretzke, 2012 [83]	2011–2	North America	Case-control	Sepsis/infection
Volpe, 2012 [84]	2010–1	North America	Interrupted time series	Antibiotics
Pakakasama, 2010 [85]	NR	Asia	Cohort	Fever
Quint, 2009 [86]	2008–11	North America	Randomized trial	Asthma
Michalowski, 2004 [87]	2011	North America	Non-RCT	Triage/patient flow
Muething, 2004 [88]	2009–11	North America	Before & after	Sepsis/infection
Melzer-Lange, 2004 [89]	2010–2	North America	Non-RCT	Pain management
Dexheimer, 2014 [90]	2008–9; 2010–1	North America	Randomized trial	Asthma
Jain, 2017 [91]	2008–9	North America	Before & after	Pain management
Fraser, 2018 [92]	NR	Asia	Cohort	Screening
Gillespie, 2016 [93]	2008–9	North America	Before & after	General PEM
Qazi, 2010 [94]	2008–10	Middle East	Cohort	Asthma
Hughes, 2013 [95]	NR	North America	Randomized trial	Screening
Meunier-Sham, 2003 [96]	2005–9	North America	Before & after	Pain management
Cunningham, 2009 [97]	2005–9	North America	Randomized trial	Screening
Einfeld, 2004 [98]	2010	Australia	Interrupted time series	Screening
Lemberg, 2005 [99]	2007–9	Australia	Before & after	Gastroenteritis
Fox, 2008 [100]	2009	NR	Before & after	Triage/patient flow

NR: not reported; PEM: pediatric emergency medicine; RCT: randomized control trial; HIV: human immunodeficiency virus.

**Fig 2:**
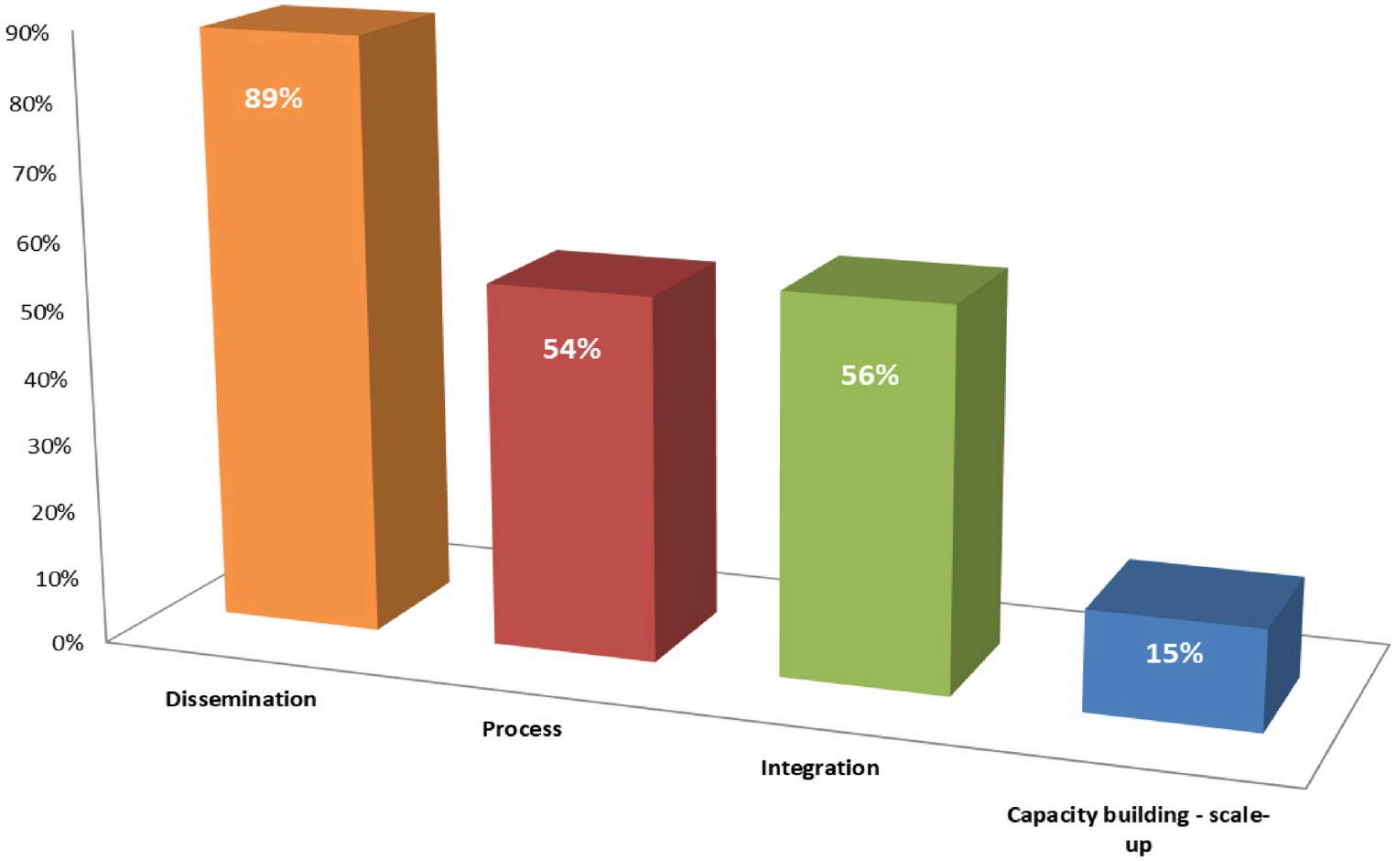
Number (%) of included studies by the type of implementation strategy.

Included studies adopted one (n = 27, 31%), two (n = 27, 31%) or three (n = 27, 31%) implementation strategies, while less than one-tenth of the studies used four strategies (n = 6, 7%). The details of the number and how these strategies were used in each included study are presented in S3 Table. Dissemination strategies were utilized by most studies (n = 77, 89%) compared to other implementation strategies; process (n = 47, 54%), integration (n = 49, 56%), and capacity building and scale-up strategies (n = 13, 15%) (Fig 2).

Studies that adopted capacity building and scale-up as part of the implementation strategies were most effective (100%) compared to dissemination (90%), process (88%), and integration (85%) (Table 2). A similar pattern of effectiveness was observed when each strategy was adopted alone. Compared to other strategies (dissemination, 92%, process, 90%, integration, 87%), the highest level of effectiveness was observed in non-randomized studies, which focused on healthcare provider-related outcomes that used capacity building and scale-up (100%) (Table 3). In contrast, the effectiveness of these strategies was low in RCTs on patient-measured outcomes.

**Table 2:** Effectiveness (%) of implementation strategies on healthcare provider-related outcomes.

Effect	Implementation strategies adopted			
	Dissemination	Process	Integration	Capacity building—scale-up
	Used in combination with other strategies			
Positive	64	36	35	13
No significant benefit	7	5	6	0
Negative	0	0	0	0
Total (n)	71	41	41	13
Effectiveness (%)	90	88	85	100
	Used alone			
Positive	17	0	2	1
No significant benefit	2	1	1	0
Negative	0	0	0	0
Total	19	1	3	1
Effectiveness (%)	89	0	67	100

n; number of studies; %, percentage of positive effect.

**Table 3:** Effectiveness (%) of implementation strategies by study design.

Effect	Implementation strategies adopted			
	Dissemination	Process	Integration	Capacity building—scale-up
	RCT studies on healthcare provider-measured outcomes			
Positive	4	0	1	0
No significant benefit	2	1	1	0
Negative	0	0	0	0
Total (n)	6	1	2	0
Effectiveness (%)	67	0	50	0
	RCT studies on patient-measured outcomes			
Positive	0	0	1	0
No significant benefit	3	1	2	0
Negative	0	0	0	0
Total (n)	3	1	3	0
Effectiveness (%)	0	0	33	0
	Non-randomized studies on healthcare provider-measured outcomes			
Positive	60	36	34	13
No significant benefit	5	4	5	0
Negative	0	0	0	0
Total (n)	65	40	39	13
Effectiveness (%)	92	90	87	100
	Non-randomized studies on patient-measured outcomes			
Positive	8	6	6	0
No significant benefit	7	5	5	1
Negative effect	0	1	1	0
Total (n)	15	12	12	1
Effectiveness (%)	53	50	50	0

RCT, randomized controlled trial; n; number of studies; %, percentage of positive effect.

On average, the effectiveness of strategies was higher in studies conducted on healthcare provider-measured outcomes versus patient-measured outcomes for both RCTs (29.3% versus 8.3%) and non-randomized studies (92.3% versus 38.3%). Reduction in the effectiveness of strategies between healthcare provider-measured outcome and patient-measured outcome, however, was higher in RCTs versus non-randomized studies. The change in the number of participants or effect estimates or both following an implementation strategy intervention in each included study is summarized in the S4 Table.

## Discussion

This review is the first systematic scoping review that identified the various implementation strategies in the emergency management of children to the best of our knowledge. Most evidence on implementation strategies came from non-randomized studies showing that dissemination strategies were most commonly used, but capacity building and scale-up strategies were the most effective implementation strategies in emergency management of children. About a third of the included studies used one implementation strategy, while two-thirds of the studies used two and three strategies, and less than 10% used four strategies. The effectiveness of the implementation strategies varied by study design and study outcome (e.g. healthcare provider-measured versus patient-measured outcomes). We observed a higher level of effectiveness of strategies in non-randomized studies that used capacity building and scale-up with a focus on healthcare provider-measured outcomes.

A timely intervention in the emergency management of children is crucial to attaining an optimal level of care. The skills and resources needed to manage children, especially in EDs require the rapid implementation of up-to-date research. Different types of implementation strategies have been used in emergency management of children [4–6], but the questions remain as to which ones are effective and how many strategies are needed in the real world.

Although dissemination strategies were most used in the included studies, capacity building and scale-up were most effective. A before and after study [9] investigated the effect of implementing a simulation-based training program on healthcare provider confidence in team-based management of severely injured pediatric trauma patients and found a positive response as the healthcare provider confidence on long-term exposure was improved. They used dissemination strategies in which healthcare providers underwent a 40-minute structured debriefing with trained debriefers after a training session. They also adopted capacity building and scale-up strategies in which various pediatric simulators and tools were used to support the implementation process to achieve a positive effect on healthcare providers.

It is crucial to identify implementation strategies, which may not produce desired results. As observed in our scoping review, a few implementation studies found no significant benefit following implementation. For example, Tavarez et al. [10] evaluated the effects of implementing e-mail-only, provider-level performance feedback on admission practice variation of physicians and reported no significant impact on management practices. They used integration strategies in which individual physician’s data/ performance was highlighted in red if it fell within the lowest quartile among all physicians and highlighted in blue if it fell within the highest quartile of performance.

The success of the implementation strategies appeared to be somewhat influenced by the study design. Our review showed that most of the included studies were non-randomized studies, and only less than one-tenth were RCTs. Arguably, the large number of study designs skewed towards the non-randomized studies may have powered the effectiveness of implementation strategies observed in non-randomized studies. That said, our review showed that the highest level of effectiveness was observed in capacity building and scale-up in non-randomized studies that focused on healthcare provider-measured outcomes. What appeared to be consistent for both RCTs and non-randomized studies was that the effectiveness of the strategies was higher in studies that focused on healthcare provider-measured outcomes versus patient-measured outcomes.

Although the ultimate goal of implementation research is to promote the overall quality of healthcare, the success of implementation strategies is in being able to influence healthcare professionals and organizational behavior positively to accept or utilize evidence-based practices [7]. Lee et al. [11] conducted a before and after study investigating the effect of implementing a clinical pathway to decrease the period of observation following the management of anaphylaxis at EDs to reduce the admission rate. They found a positive effect on the overall admission rate, which was reduced from 58 to 25% following the implementation. While they reported a positive outcome on healthcare provider-measured outcomes, they found no benefit on the patient-measured outcome (percentage of patients that returned to the ED within 72 hours) following the implementation. Their findings and that of other included studies in this review showed that perhaps the focus of implementation studies on patient-measured outcomes may not be a good marker of a successful implementation strategy. We also observed that the reduction in the effectiveness of strategies between healthcare provider-measured outcomes and patient-measured outcomes was higher in RCTs than non-randomized studies. This suggests that the effectiveness of the implementation strategies may be exaggerated in non-randomized studies.

The strengths of this review include using an a priori protocol that followed the standard accepted methods for scoping reviews, and reporting according to the PRISMA Extension for Scoping Review guidelines. The inclusion of a multidisciplinary team, including experienced systematic reviewers, experts in implementation science, clinical epidemiology, and pediatric emergency management, provided adequate guidance to the reviewers during study selection, data extraction, and interpretation of the results. Our study is not without limitations. Most of the included studies were from North America; thus, worldwide generalizability of our results may be difficult because of cultural variations, which may affect behavior towards implementing research evidence. We did not appraise the risk-of-bias of included studies, which is in keeping with scoping review methods [17]. Because most of the included studies were non-randomized studies with possible exaggeration of effectiveness of strategies, we acknowledged that more robust implementation RCTs with sophisticated methodological approaches are needed to accept or refute our findings. We only performed descriptive statistical analysis, which is consistent with our a priori protocol. Although we searched multiple bibliographic databases for completeness of the search, we acknowledged that we may not have captured all relevant studies due to our inclusion criteria.

Further research is needed to determine barriers to adopting other implementation strategies that appeared to be more effective but not commonly used. More data is also required to determine the optimal time to implement these strategies and their long term effects. Our scoping review has helped summarize the available evidence on implementation strategies in emergency management of children and highlighted the characteristics of successful ones.

In conclusion, studies on implementation strategies in emergency management of children have mostly been non-randomized study designs with possibly exaggerated effect sizes. Better study designs such as RCTs should be conducted more frequently when comparing implementation strategies. This review suggests that dissemination is the most common strategy, and capacity building and scale-up strategies are the most effective strategies.

## Supporting information

S1 Table PRISMA extension for scoping review checklist. (DOCX)

S2 Table Search strategy. (DOCX)

S3 Table Summary of the number and type of implementation strategies used in the included studies. (DOCX)

S4 Table Effect of implementation strategies on the number of participants or effect estimates measured in the included studies. (DOCX)

S1 Fig Number (%) of included studies by study design. RCT, Randomized controlled trial.
(TIFF)
