# Supplementary material for: Implementation strategies in emergency management of children: A scoping review
Source: PLoS One. 2021 Mar 24;16(3):e0248826. doi: 10.1371/journal.pone.0248826 (PMC7990517; doi:10.1371/journal.pone.0248826)
Supplement: S1 Table — (DOCX) [file pone.0248826.s001.docx]

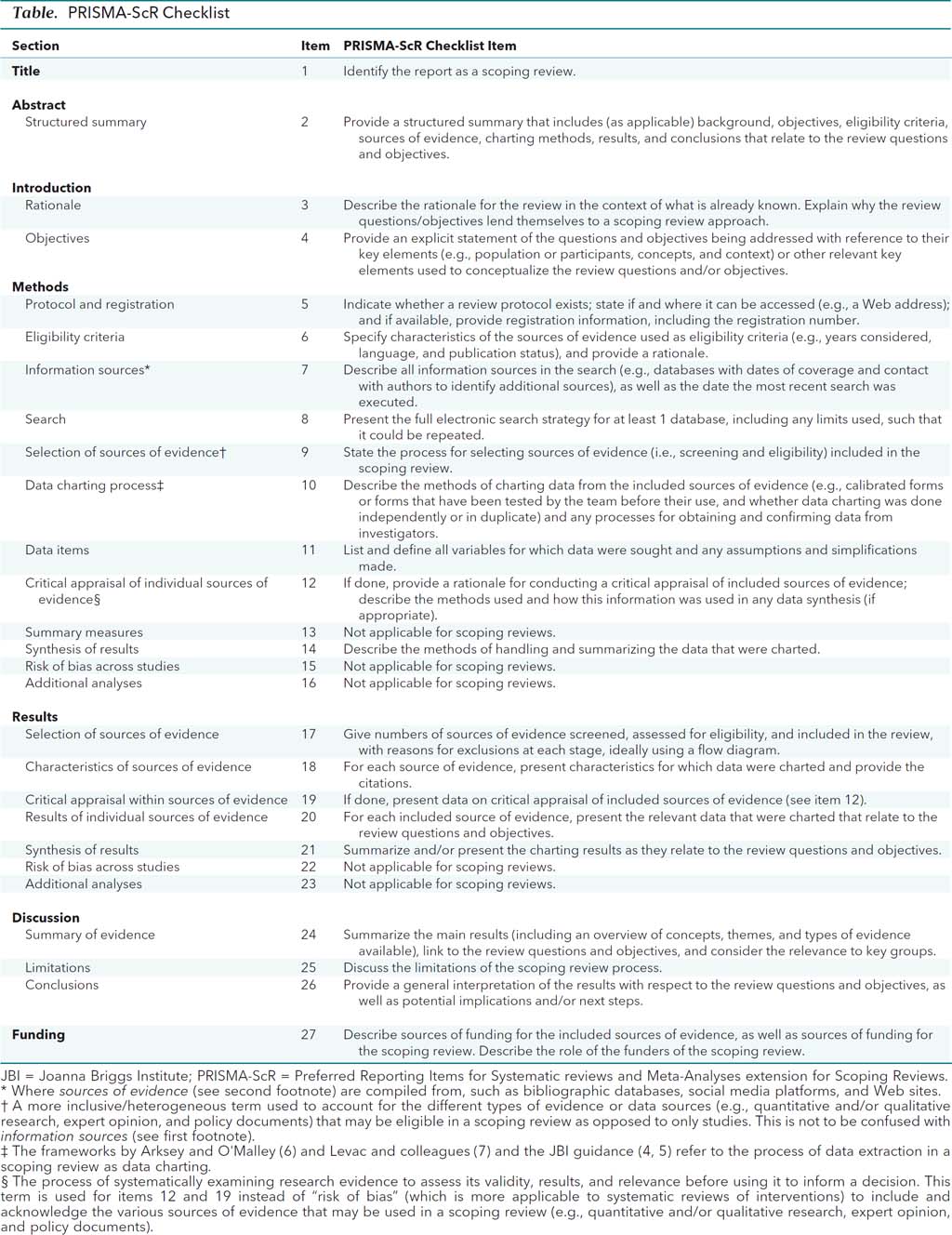


From: Tricco AC, Lillie E, Zarin W, O'Brien KK, Colquhoun H, Levac D, et al. PRISMA Extension for Scoping Reviews (PRISMAScR): Checklist and Explanation. Ann Intern Med. 2018;169:467–473. doi: 10.7326/M18-0850.
