# Supplementary material for: Implementation strategies in emergency management of children: A scoping review
Source: PLoS One. 2021 Mar 24;16(3):e0248826. doi: 10.1371/journal.pone.0248826 (PMC7990517; doi:10.1371/journal.pone.0248826)
Supplement: S3 Table — (DOCX) [file pone.0248826.s003.docx]

| S3 Table. Summary of the number and type of implementation strategies used in the included studies | | | | | | | | | | | | | | |
| --- | --- | --- | --- | --- | --- | --- | --- | --- | --- | --- | --- | --- | --- | --- |
|  |  | | | **Dissemination** | | | **Process** | | **Integration** | | **Capacity building and scale-up** | | | |
| **First author, year of publication** | **N strategies** | | | **Used** | | **Notes** | **Used** | **Notes** | **Used** | **Notes** | **Used** | | | **Notes** |
|  |  | | |  | |  |  |  | | | | | | |
| McGrew,  2018^[8]^ | | 2 | | | x | The practice guidelines were made available to all practitioners who routinely participate in the care of pediatric trauma patients. Internal education sessions were performed at combined division meetings with Trauma, PICU and PED faculty. A grand rounds presentation was also given for all general surgery residents describing the guideline and the literature behind it and similar presentations were given to pediatric and emergency medicine residents. Posters depicting the guidelines were hung conspicuously in areas in our TC and the guidelines were included in our institutions’ pediatric trauma packet, which contains documents used in the pediatric trauma evaluation and resuscitation. | x | The guidelines were proposed and discussed in a multidisciplinary trauma department meeting, which included trauma surgeons, pediatric emergency physicians and PICU physicians, where they were accepted and adopted for use in our TC. | | | | | | |
|  |  | | |  | |  |  |  | | | | | | |
| McLaughlin, 2018^[9]^ | | 2 | x | | | Simulations most frequently occurred near the summer to coincide with new hires and to avoid times of peak ED volume (e.g., influenza season). To preserve fidelity, simulation-based training sessions were not pre-announced and were paged out as actual level I trauma activations. These simulations were carried out in situ in the trauma bay, with sessions conducted during daytime and overnight shifts. Predetermined criteria to terminate the simulation included a coincident actual trauma activation or high surge capacity in the ED. After each simulation-based training session, participants underwent a 40 min structured debriefing with one of two trained debriefers. Debriefing used the advocacy-inquiry method to focus on teamwork and team function through a reflective learning process. | | | | | | x | Each simulation-based training session used one of the following high-fidelity pediatric simulators: SimBaby™(Laerdal Medical, Stavenger, Norway), Gaumard HAL S3004 pediatric simulator (Gaumard® Scientific, Miami,FL), or TraumaChild (Simulab Corporation, Seattle, WA) combined with the Laerdal SimBaby™ software and VS monitors. These are tools to support implementation process. | |
| Tavarez, 2017^[10]^ |  | | |  | |  |  |  | x | During the intervention period (fiscal year 2011), we sent quarterly reports via e-mail to each PEM physician. So that the individual physician could compare his or her performance to that of his or her peers, the individual physician’s data were highlighted in red if it fell within the lowest quartile among all physicians and highlighted in blue data if it fell within the highest quartile. Data representing the most extreme values (highest or lowest) for all physicians were circled in red. | | | | |
|  |  | | |  | |  |  |  | | | | | | |
|  |  | | |  | |  |  |  | | | | | | |
| Lee,  2018^[11]^ | 3 | | | x | | A hospital-wide announcement was made on the intranet and by internal e-mail to alert providers to this update (of the pathway). ED clinicians participated in several joint educational sessions to review the pathway and diagnostic criteria for anaphylaxis. Triage protocols were reviewed with nursing staff. Progress data was shared at multidisciplinary division meetings, included in the weekly RN updates and posted on the ED Quality Board and on ED screensavers. | x | Multidisciplinary team (physicians, nurse practitioners and RNs from the divisions of PEM and Allergy, as well as including an improvement adviser and data analyst from the Office of Clinical Quality Improvement) reviewed existing literature to discuss revisions to existing pathway. Evidence was also reviewed with all clinicians for further input. | x | We highlighted on the pathway screen that the first-line treatment of anaphylaxis is epinephrine, delivered intramuscularly. To emphasize epinephrine as the first-line therapy, a chart outlining the diagnostic criteria previously established by expert consensus, was placed on the main pathway screen. Review of anaphylaxis cases and discussion of feedback from ED bedside providers. Team assessment (physicians and nurses jointly evaluating patients brought back immediately from triage) was stressed for anaphylaxis patients. Front-line ordering clinicians, as well as fellows and attending physicians, were encouraged to immediately order epinephrine by using the anaphylaxis order set, so that the nursing staff could expeditiously retrieve and administer the medication. | | | | |
| Waddell, 2014^[12]^ | 2 | | |  | |  | x | The guideline was introduced to all ED nursing staff through nursing in-service education. The ED nurses were provided with a step-by-step approach to the use of the guideline in the treatment of varying degrees of mild, moderate and severe dehydration in children. Education of the guideline was also given to the ED medical staff. | x | Increased knowledge and awareness in the treatment of dehydration in children was also highlighted and further discussed to ensure that these patients were receiving the appropriate triage categories and interventions in a timely manner. | | | | |
|  |  | | |  | |  |  |  | | | | | | |
|  |  | | |  | |  |  |  | | | | | | |
| Johnson, 2018^[13]^ | 3 | | | x | | The asthma CPG was implemented via an electronic inpatient order set, publication of the CPG with a reference packet on the hospital intranet for providers. | x | An asthma specific ED order set was developed. | x | In-person and electronic education by Committee members. | | | | |
| Puffenbarger, 2018^[20]^ | 2 | | | x | | Implementation of the Pediatric Closed Head Injury Assessment Tool was achieved through provider education sessions. | x | Head CT use was monitored for 12 months post-implementation as a form of process and outcome evaluation. | | | | | | |
| Lukes,  2019^[21]^ | 3 | | | x | | Ongoing education of provider and ED nurses regarding QI interventions. | x | Baseline data evaluation to inform choosing of evidence-based interventions. | x | Improving provider workflow by creating an order set to promote accessibility of evidence-based orders, decrease delay and promote patient safety. Changing workflow by providing order set that established the administration of broad-spectrum results first, before complete blood count results. Creation of triage order set which required triage of all patients to see provider within 10 min. | | | | |
| Carson,  2018^[22]^ | 2 | | | x | | Twenty-min educational session on child physical abuse was offered to all ED HCPs during two regularly scheduled staff meetings. The educational sessions were delivered in person and included an evidence-based overview of child physical abuse; types of injuries that raise the index of suspicion for abuse; use of the validated Escape Instrument; the specific steps in the systematic screening protocol; and documentation of findings, including diagnostic coding of suspected and confirmed child physical abuse. | | | x | Laminated copies of the screening protocol, Escape Instrument and child physical abuse diagnostic codes were placed in the physicians' rooms and at the nurses’ station. All ED HCPs were given laminated badge cards containing the Escape Instrument and diagnostic codes. Finally, laminated reminders with the words “Did you remember to ESCAPE?” were placed on each computer screen in the patient rooms and at the nurses station to remind the emergency nurses to complete the Escape Instrument during patient intake; completion of the Project Evaluation Survey was independent of the pre-test/post-test and educational session. | | | | |
| Libetta,  1999 ^[23]^ | 2 | | | x | | All medical staff in the accident and ED department were given a lecture on the rules and their implementation before the start of the study period. | | | x | Each patient who then presented with an appropriate injury had a form depicting the Ottawa ankle rules attached to their notes by the triage nurse that the doctor could then use as an aide memoire when deciding whether radiography was required. | | | | |
| Hendrickson, 2018^[24]^ | 2 | | | x | | The E-CDS was launched in the electronic health record throughout the hospital. Providers were introduced to the order set in formal (grand rounds) and informal training sessions conducted by study investigators. | x | A CPG for the evaluation of children with suspected appendicitis was developed by a multi-disciplinary group of PEM physicians, radiologists and surgeons from the children’s hospital. Clinical content was based on the low-risk appendicitis rule and the pediatric appendicitis score. The guideline content was built into a decision support order set, the E-CDS. | | | | | | |
| Norton,  2007^[25]^ | 1 | | | x | | Staff training was done before CP implementation. | | | | | | | | |
| Dona, ^[26]^  2018 | 1 | | | x | | Three CP training sessions were presented to PED and Pediatric Acute Care Unit physicians and residents along with an overview of the guidelines, the rationale behind the treatment. | | | | | | | | |
| Mohan,  2018^[27]^ | 2 | | |  | |  |  |  | x | An electronic history and physical examination template was created for the evaluation of children with chest pain prompting physicians to check for specific signs and symptoms and document cardiology consultation. A mechanism for follow-up was instituted in computerized order entry system if the ED provider felt cardiology follow up was warranted. | | x | The pathway was implemented with several weeks of intensive education efforts aimed at ED and cardiology faculty, as well as ED and pediatric housestaff and nurse practitioners. | |
| Jones,  2017^[28]^ | 1 | | |  | |  | x | The advice of a health-care stakeholder group was part of the Shorter Stays in EDs National Research Project. | | | | | | |
| Murray,  2017^[29]^ | 1 | | | x | | Before the pathway implementation, all ED staff were briefly educated during a departmental meeting on the pathway specifics and goals. | | | | | | | | |
| Geurts,  2017^[30]^ | 2 | | | x | | The guideline could be retrieved from the protocol server of the Erasmus Medical Center website on initiative of the clinician. Creating an optimal environment for implementation of the decision support system, we created group lectures for nurses at the start of their shift, repeated individual briefings and reminders by posters, email and newsletters periodically. The implementation process was closely monitored and evaluated. | | | x | The clinical dehydration scale and current guidelines on treatment of acute gastroenteritis were incorporated in an electronic, easily accessible CDSS, available at each desk top at the ED. | | | | |
| Ahmad,  2017^[31]^ | 3 | | | x | | The final version of the guideline was disseminated via email to all pediatric residents, fellows and faculty at St. Louis Children’s Hospital on February 20, 2013 and was simultaneously made available on the hospital intranet site along with other clinical guidelines used in the ED. | x | The guideline was created by the authors after an extensive review of the literature and consultation with experts in emergency medicine, neurology, infectious disease and critical care at our hospital, as well as infectious disease specialists at other hospitals. It was approved by physician, nursing and administrative leadership of the hospital before implementation. A departmental conference was conducted to review a draft of the guideline. | x | Electronic prompts in the ED EMR reminded providers to review the guideline when ordering any HSV polymerase chain reactions or IV acyclovir for patients who were 42 days or younger. The prompts reminded clinicians of the shortage and to ensure their patient met criteria for testing and treatment. | | | | |
| Gildenhuys, 2009^[32]^ | 3 | | | x | | The Area Health Service guidelines project officer introduced the final CPGs worksheet to ED staff at educational sessions. | x | The Area Health Service guideline project officer’s role also involved mentoring and regular communication with ED staff providing pediatric emergency care. The project officer sought feedback and incorporated changes into the asthma CPGs. | x | An Area Health Service guideline project officer was employed to coordinate the implementation of the asthma CPGs at the hospital and in the ED. This officer also reviewed use of the CPGs worksheet and reinforce behavior of using the CPGs worksheet. In conjunction with local parties and a local champion, the CPGs were adapted into an asthma CPGs worksheet. | | | | |
| Rutman,  2016^[33]^ | 3 | | | x | | Literature review and grading of evidence for selecting potential EBIs (to modify the pathway). | x | In addition to organizational changes, such as renaming the order sets to make them easier to find and use, the pathway was modified to target specific opportunities for improvement. | x | For two weeks before implementation, the pathway modifications were discussed at ED and inpatient provider meetings. E-mail notifications (including to physician and nurse job aids) were sent. In addition, a mandatory web-based training module was distributed. This training was required for all ED and inpatient providers. It described all pathway modifications and included a knowledge assessment with a required minimum passing score. Finally, laminated copies of the pathway were placed outside patient rooms and in provider work areas to ensure visibility and access. | | | | |
| Lin,  2016^[34]^ | 1 | | | x | | Triage nurses completed a two‑month CPETS training course (120 instructional hours) before using the CPETS and associated software in the pediatric emergency room. | | | | | | | | |
| Shah,  2016^[35]^ | 2 | | | x | | Before implementation of the diagnostic algorithm, it was presented at departmental/divisional meetings in pediatric surgery, emergency medicine and radiology for consensus approval. | | | | | | x | Implementation took place after aggressive education efforts within pediatric surgery, emergency medicine and radiology. | |
| Dandoy,  2016^[36]^ | 3 | | | x | | Intense port access education, along with the F&I process module, was given to all ED and CBDI clinic staff with interval refresher courses. | x | Team comprised of stakeholders reviewed the current antibiotic administration practice and process map, gaps in practice and identified areas for improvement. They defined potential interventions and their level of reliability and tested each by using PDSA cycles to enable improvement strategies to be tested and refined. They identified barriers (delays in central line access, delays in ordering of antibiotics, delays in rooming of patients/room availability, variable awareness of patient arrival to ED, lack of a standard team approach to patient evaluation and antibiotic administration). | x | The ED physician used a standardized order set that was completed at the time of the referral to place orders for labs and antibiotics. A patient room was identified and made available in anticipation of the patient’s arrival. Antibiotics and lab slips that were ordered prior to arrival were placed in the room along with the proper supplies to access the patient’s line. A team page was made at time of the referral as well as to alert the physician and charge nurse that an F&I patient would arrive soon. Finally, the ED team created a pre-arrival huddle between the lead physician, nurse and emergency medical technician (F&I team). This team would review the checklist, verify the placement of orders and verify the proper supplies were obtained and room was reserved. The ED Referral Coordinator confirmed orders were placed within 10 min of referral, completed a reminder card for the greeter desk in the ED with the patient’s name. Once the patient arrived in the ED, the EMR automatically sent an arrival page to the referral group noting the patient had arrived. The Charge RN or designee then escorted the patient to the held room. Central line access supplies were available in the room as well as the appropriate antibiotics. If a patient was anticipated to have port central venous catheter issues in the past, the VAT was called upon arrival to assist in vascular access. Posting process run-charts in the QI board in the ED and clinic made awareness of performance. Results were also placed in the ED and CBDI dashboard for monthly analysis by leadership. All failures were evaluated in real time. Nurses, physicians and family members were contacted shortly after the encounter and learnings were analyzed and implemented when indicated. Implementation of rapid response team in Sept 2014: The rapid response team, consisting of the ED F&I team plus a VAT member, prioritized the initial assessment of the F&I patient upon arrival in the ED. The VAT was alerted of the anticipated time of the patient’s arrival when the call was made to the ED. Upon arrival into the ED, a rapid response pager alerted the team that the patient had arrived and they would evaluate their status in a timely manner. | | | | |
| Cohen,  2016^[37]^ | 2 | | | x | | ED nursing staff education and training on obtaining central venous access. ED nursing and triage staff were also educated on the importance of rapid triage to decrease patient wait times after triage check-in. ED nursing staff was educated on the protocol prior to initiation and received continuing yearly re-education. | | | x | Quarterly results of the median time to antibiotics for this patient population were shared at faculty and staff meetings to encourage improvement and adherence to the protocol. Pre-approved order sets were implemented that allowed nursing staff to begin obtaining central access and blood work without the requirement of a physician order. | | | | |
| Fallon,  2015^[38]^ | 3 | | | x | | The Appy-Score system and reporting templates were presented at a faculty meeting and via e-mail and feedback and suggestions were elicited. | x | The templates were built with input from all radiologists within the body division of the Department of Pediatric Radiology (19 radiologists) and four versions of the templates were tested for clarity, ease of reporting and inclusiveness of all pertinent findings. Six separate structured reporting templates were created, one for each Appy-Score stratum. Final versions of the reporting templates provided the patient’s Appy-Score as well as a summary of the findings that led the radiologist to arrive at the score. After consideration of feedback and suggestions, the scoring systems and reporting templates were finalized and department leadership mandated implementation. | x | In order for the Appy-Score to be reported consistently and clearly, structured reporting templates were created. Template was integrated into the routine workflow of the radiologists. | | | | |
| Jeong,  2015^[39]^ | 3 | | | x | | We informed the emergency physicians and nurses about the five clinical practices on July 2, 2012. Education sessions were held once a week to teach the practices, such as teaching about medical errors with returned patients. | x | A multidisciplinary team composed of emergency physicians, pediatric emergency physicians and nurses convened once a week to develop clinical practices over two months. Literature was reviewed to identify the classification and reduction methods for return visits. Two approaches were used to develop practices. First, we surveyed emergency physicians and nurses at the ED about causes of return visits. A diagram on each management step in the ED was used to select steps related to return visits. The most problematic issues were discussed and methods for reducing return visits developed. Review of medical record data and analysis to determine factors involved in return visits. These were used to inform the development of five clinical practices. | x | Multidisciplinary team members analyzed return patients daily after application of the clinical practices. If there were medical errors, we used two approaches. One was to notify the involved physician directly about the errors. The second was to discuss it at morning conference to prevent the same errors from other physicians. Morning conference is held five times a week. The patients were managed by emergency physicians supervised by board-certified emergency attending physicians. If emergency physicians judged that the patients required admission, they consulted physicians in other departments using the EMR system. | | | | |
| Dexheimer, 2014^[40]^ | 1 | | | x | | Two months prior to the study: 1) physicians were informed about the study in the operational emergency management, faculty and monthly resident meetings; 2) an email from the ED director (division chair) describing and supporting the study was sent out to the ED staff; 3) respiratory therapists were informed during their monthly management meetings; and 4) for a week prior to the study the nurse leadership informed the nursing staff through the twice-daily meetings before the start of each shift. At all of these meetings, an investigator explained the study and answered any questions that arose. | | | | | | | | |
| Higginbotham, 2014^[41]^ | 2 | | | x | | The guideline was made available to ED physicians who were responsible for initiating the protocol and consulting trauma services for further evaluation and management. | x | Guideline for the evaluation of suspected non-accidental trauma was developed on the basis of available literature. | | | | | | |
| Geurts,  2014^[42]^ | 2 | | | x | | A targeted and multi-faceted implementation strategy including instructions concerning the guideline on group and individual level adopted from implementation theories. Before and during the course of the study, we gave several group lectures in medical staff meetings to the pediatric staff and residents. Also, this was done at the ED to the attending medical and nursing staff at every shift for the period of one month. Laminated pamphlets of the guideline were available at the ED. | | | x | Finally, written instructions were sent three times to all HCPs working at the ED during the implementation period by email as a reminder. | | | | |
|  |  | | |  | |  |  |  |  |  | | | | |
| Boutis,  2013^[43]^ | 3 | | | x | | Emergency physicians underwent a teaching session and were provided with pocket cards that outlined the use of the ankle rule. Wall posters reviewing the rule were displayed in the ED and charts of children with ankle injuries were flagged. Physicians were trained to manage all low-risk ankle injuries in children (with or without radiography) with a removable immobilization device (e.g., brace), crutches as needed for pain and return to activities as tolerated. | x | Use of Low Ankle Rule was based on knowledge of barriers to implementing strategies, available evidence and consultation with knowledge transfer experts. | x | Physician champions were identified at each site. Computerized decision support system for physicians to enter key clinical variables that would automatically generate the recommendation of the ankle rule for radiography. This system was not linked to ordering of ankle radiographs nor was it a requirement to complete clinical care. | | | | |
| Taylor,  2013^[44]^ | 1 | | | x | | The algorithm was displayed in poster format in all triage areas and pediatric patient care work areas. Pediatric NIAP credentialing required Pediatric Advanced Life Support certification and approval to work in the ED triage area. The ED pharmacists also presented a package of NIAP learning material (a PowerPoint presentation, the NIAP algorithm and the Standing Order) during in-services to the ED nursing staff. The learning material was also available online for subsequent access. All nurses subsequently undertook an online examination that consisted of seven multiple choice and three long-answer questions related to specific patient scenarios. | | | | | | | | |
| Russell,  2013^[45]^ | 2 | | | x | | Before the introduction of this pathway, a formal education process was conducted for both the pediatric surgeons and the PEM physicians. Aggressive educational push aimed at both the pediatric surgeons and PEM providers to increase awareness and compliance with the new guidelines during the 12 months after pathway implementation. | x | A multidisciplinary team including pediatric surgeons, PEM physicians and pediatric radiologists met at the Medical University of South Carolina Children’s Hospital to create a pathway to formalize the evaluation of pediatric patients with abdominal pain concerning for appendicitis. | | | | | | |
| Hack,  2013^[46]^ | 1 | | |  | |  |  |  | x | The investigator of the study did assist in the implementation of the routine, rapid HIV screening serving as a liaison between the physicians and nurses and the New Jersey Department of Health and Senior Services HIV counselors, encouraging physicians and nurses to offer HIV screening. | | | | |
| Wolff,  2012^[47]^ | 3 | | | x | | The educational component consisted of a didactic session at a departmental meeting for physicians and nursing leadership and separate online education modules for physicians and nurses. The education reviewed the evidence behind the recommendations and highlighted key changes in practice. The CP was posted on our institutional intranet and served as an educational tool with hyperlinks to information such as the nomograms for phototherapy and exchange transfusion, recommended laboratory studies and standardized discharge instructions. | x | Compliance and performance data were shared with the ED staff at regular intervals after pathway implementation. | x | The educational effort was reinforced by enlisting the help of several nurses and physicians to become informal peer champions of the project, providing support and real-time feedback to colleagues when working in the ED. | | | | |
| Doyle,  2012^[48]^ | 1 | | | x | | All ED nursing staff members (more than 60 nurses) were trained in ESI during a six-week period. Training included the Emergency Nurses Association’s didactic content modified for pediatrics, followed by practical orientation with a clinical nurse specialist in triage using the ESI. After training was complete, chart audits were conducted with each triage nurse to assess correct application of the ESI algorithm. Guidelines for triage were standardized and all nursing and physician staff were educated. | | | | | | | | |
| Waseem,  2012^[49]^ | 3 | | | x | | In the ED, huddles among physicians, nurses and administrators were held frequently throughout the day. It was during these briefings that written updates were provided to the physicians as information and recommendations regarding testing and treatment became available. | x | Hospital emergency incident command system was activated which allowed coordination of all resources and engaged all involved services, including infection control, laboratory, pharmacy, information management, admitting and clinical departments and others who also participated in daily conference calls with the New York City Department of Health. It also allowed for brainstorming and ownership and offered help and solutions from within the hospital. | x | Increase in service hours of existing staff and increase in nurse staffing; Revisions to medical record systems: "Oseltamivir and zanamivir were added to the electronic pharmaceutical dispensing system located in the ED so that uninsured patients and patients seen after the hospital pharmacy or neighborhood pharmacies were closed could receive Treatment before discharge from the ED and the EMR was amended to simplify the mandatory fields on the triage screen for the nurses and to create a single-page physician disaster note. Physicians were assigned only a few patients at a time by the charge nurse who was monitoring the flow of patients. Placing all the charts in the physician rack was thought to be overwhelming for the physicians. | | | | |
| Hendrickson, 2012^[50]^ | 3 | | | x | | The trauma MTP committee educated others in the hospital utilizing multiple media types. | x | A trauma MTP committee was formed on each of the institution’s two campuses, consisting of a pediatric surgeon, a pediatric anesthesiologist, a PED physician, a pediatric hematologist, a pediatric intensive care physician, two pediatric transfusion medicine physicians, the campus-specific blood bank supervisor, the blood bank manager and the lead pediatric nurse trauma specialist. This committee developed and refined the MTP. | x | The trauma team was encouraged to notify the blood bank initially by phone of all potential MTPs, such that products could be prepared in advance. An MTP panel of lab tests was included in the protocol and tubes for MTP lab collection were attached to all blood coolers as a reminder to clinicians and orders for all subsequent MTP labs were automatically placed by the laboratory under the authorization of the initial ordering physician. | | | | |
| Crocker,  2012^[51]^ | 1 | | | x | | Efforts were made to increase pain management awareness with techniques woven into the fabric of the ED via education; protocols; and, importantly, changes in attitudes toward pain control. Collectively, this process is referred to as the "Comfort Zone.” Extensive nursing and physician education was provided regarding the implementation of the pain protocol. | | | | | | | | |
| Angoulvant, 2012^[52]^ | 3 | | | x | | Scientific discussions among emergency physicians, residents and specialists in pediatric infectious diseases. Local guidelines were available through physician pocket guides and the hospital intranet beginning in October 2006. Rapid antigen diagnostic tests for group A streptococcus and influenza were available in the PED and their use was strongly encouraged. | x | Each PED physician was instructed to follow the guidelines for diagnosing and treating acute respiratory tract infections. | | | | x | Teaching sessions for new PED and pediatric residents were performed twice a year. | |
| Larsen,  2011^[53]^ | 3 | | | x | | An educational program for the ED physicians, nurses and technicians regarding the national pediatric septic shock guidelines, including the need for timely clinical interventions and the potential impact on mortality rate, was initiated. In February 2007, the tool (a one-page document) was displayed in the ED triage nurses’ station and in the protocol drawer. On an approximately weekly basis during the first month of implementation, the ED nurse clinical specialist (Ms. Mecham) and ED physician (Dr. Greenberg) spoke to the ED nurses and physicians regarding the triage tool and guideline. After monthly case review, Ms. Mecham identified representative or problematic cases and reviewed details of triage delays, missing VSs, errors in triage, management issues, or exceptional work with the individual nurses involved. The lead ED physician (Dr. Greenberg) provided periodic feedback about individual cases at monthly ED staff meetings. One project barrier that was identified early was the lack of ED point-of-care lactate testing. Support was solicited from the chief of pathology/laboratory medicine and the central laboratory technicians to make necessary supplies available in the ED so that serum lactate could be analyzed from the same sample drawn for blood gas and electrolyte testing. Approval for ED bedside lactate measurements occurred in January 2007. Another identified project need was a study coordinator and administrative help to access patient records, electronic data and chart review and to generate reports. PCMC provided a study coordinator for the first two years (2007–2008). | x | Multidisciplinary team reviewed existing national guidelines and developed a reference tool that defined abnormal age-appropriate VSs and physical findings and a care guideline for patients with suspected septic shock. | x | Salary support and responsibilities as study coordinator shifted to the project nurse clinical specialist (Ms. Mecham) thereafter. Within the PCMC Division of System Improvement, data were collected and stored to facilitate quarterly reports for hospital executive meetings and to identify barriers. Data were maintained within the PCMC Division of System Improvement and provided to the ED staff through both individual feedback and annual reports. | | | | |
| Cruz,  2011^[54]^ | 4 | | | x | | After creation of the protocol, a communication strategy was developed. The month before protocol initiation, two-hour education sessions were conducted with all ED nurses and the transport team to explain inclusion criteria and changes from existing procedures. This education was repeated four months later. E-mail communications were sent to ED and PICU staff and leadership was available to answer questions. Subspecialty services were involved in protocol design to verify acceptable fluid volumes, empiric antibiotics and pertinent laboratory evaluation. | x | Root-cause analyses and morbidity and mortality conferences revealed areas for improvements in sepsis management. A multidisciplinary team identified several obstacles including variation in experience of staff in performing initial evaluations; lack of adequate nursing staff for resource-intensive patients; difficulty obtaining frequent vital-sign measurements; lack of standardization of empiric antibiotics and diagnostic tests; lack of medication priorization; and barriers to patient flow through the institution. | x | As a continuous QI project, it was recognized that modifications would be necessary and communicated. With feedback, the order set was revised to include additional medications that were commonly used in patients with shock, add laboratory measures and change empiric antibiotics for previously healthy children. These changes and interval outcome measures were posted in the ED and e-mailed to providers and stakeholders every two months. Subsequently, a prospective QI project was designed to measure the impact of early recognition and intensive nursing resources on the ability to deliver fluids and antibiotics more rapidly to children in shock; the project was termed the “shock” protocol. If VSs were outside of age appropriate norms, an electronic alert forced the triage nurse to consider the shock protocol. With activation, the transport team and PICU charge nurse were also alerted of a potential admission. | | x | A graphical flow sheet was created by the team to assist in ongoing assessments and facilitate handoffs across the continuum of care. Information technology helped create a computerized triage tool that corrected heart rate for pyrexia. | |
| Iyer,  2011^[55]^ | 4 | | | x | | Data presentations to staff demonstrating that interventions directed at a single key driver were insufficient to achieve our goal were vital in convincing them that a larger, more fundamental system change was required. | x | On the basis of a review of the literature, interviews with key stakeholders, expert consensus and reviews of isolated examples of patients receiving timely analgesics, a multidisciplinary improvement team identified a set of operational factors, or key drivers, believed to be critical to the performance of appropriate initial pain management for children presenting to the ED with acute extremity injury. These key drivers focused the development of an intervention. Proposed goals were vetted with institutional leaders. Existing processes for pain assessment and analgesic delivery were studied and initial improvement protocols were pilot tested using a PDSA cycle methodology. These informed the development of the "orthopedic evaluation process" which simultaneously addressed all four key drivers. | x | Dissemination of run-chart data in near real time allowed the team to provide feedback to emergency-department staff and reinforce education on process changes. These next-day failure analyses enabled staff to learn rapidly about our intervention design and understand the subpopulation of children it was meant to address. | | x | Technical support was provided by a QI consultant and data analyst. | |
| Fagbuyi,  2011^[56]^ | 2 | | |  | |  | x | In response to H1N1-associated surge in patient flow in the previous season, they projected the need for a process that would incorporate the following elements: identification of additional clinical space for rapid screening, the use of a paper chart rather than an EMR, the ability to provide discharge teaching to multiple families concurrently and the use of preprinted discharge instructions and prescriptions. | x | They developed paper-based forms and identified office space adjacent to the ED to be used for the rapid screening process. This space was designated as the rapid screening unit and consisted of six partitioned office cubicles and a desk for a physician and nurse team to chart. Staffing was provided by the use of a combination of strategies: redeployment of already present staff and telephone calls using our on-call list and ED backup providers not scheduled to work. Using a paper checklist, the physician completed a brief history, confirmed the absence of chronic underlying disease and performed and documented a physical examination, including items aimed at detecting the most common complications of influenza. Physicians were instructed not to routinely examine the ears or throat unless the patient complained of pain or was too young to do so. Physicians were provided with a written algorithm to help them decide which patients should receive oseltamivir. The discharge instructions and the prescriptions were preprinted and the prescriptions had checkboxes for age/weight-based dosing, formulation and duration of therapy | | | | |
| Fein,  2010^[57]^ | 2 | | | x | | Research staff gave several in-service talks to ED physicians, nurses, nurse practitioners and technicians about the purpose and logistics of the Behavioral health screening-ED system’s content and procedures. | | | x | In response to staff feedback during the feasibility phase, we designed and implemented a web-based, searchable resource database of regional mental health providers. Research staff verified all of the mental health providers’ data by telephone. To access the database, a staff member would enter data on a patient’s mental health needs, type of insurance and residency (i.e., county) into the computer and the program generated a list of several local mental health providers with contact information. | | | | |
| Babl,  2010^[58]^ | 2 | | | x | | The comprehensive and multidisciplinary program includes a standardized sedation checklist, parent handout and staff education materials, including a manual, lectures, a multiple choice test and staff competencies and is taught by nurse educators. | x | An assessment of the quality and safety of pediatric procedural sedation training and credentialing program was conducted six months after implementation and showed significant improvements in important proxy markers of sedation safety including risk assessment, monitoring and documentation. | | | | | | |
| To,  2010^[59]^ | 1 | | | x | | PAMG introduced to all health care providers (including physicians, nurses, residents and respiratory therapists) at the ED of The Hospital for Sick Children in Toronto, Canada after January 2003 through multiple presentations at their rounds and business meetings. Methods of disseminating PAMG included: 1) providing an ample supply of the reminder card for use at the ED; and 2) distributing individual packages (with a short description of the study and the reminder card) to the ED staff in their mailboxes. | | | | | | | | |
| Trottier,  2010^[60]^ | 1 | | | x | | Before its implementation, this protocol was presented, discussed and approved by the physicians. | | | | | | | | |
| Cruz,  2010^[61]^ | 4 | | | x | | E-mail updates were sent daily to inform the Houston medical community on the most recent local epidemiologic data, screening recommendations and updated Centers for Disease Control and Prevention recommendations. Effective communication between physicians in the hospital and community, as well as between physicians and families, was essential to decrease anxiety and unnecessary patient visits. Efforts were also made to address health care worker anxiety and involved online modules and videos available on the hospital intranet site to educate staff about H1N1. All clinical staff members and ancillary staff were fit-checked for N95 respirators, which were readily available throughout the hospital. | x | Surge capacity was augmented both through utilization of existing institutional resources and by creating a novel area in which to treat patients with potential airborne pathogens. Infection control procedures and patient safety were also maximized through patient cohorting and adaptation of social distancing measures to the ED setting. The decision to implement the MPERT required monitoring trends of patient volumes and other factors. | x | At the onset of the study, patients were being liberally screened for influenza. Three hundred sixty-nine rapid influenza assays were performed in the week preceding MPERT, 3.5% (13) positive for H1N1 and 680 were performed during the MPERT week, 1.6% (11) positive for H1N1). Despite extensive screening, only 2.8% (18) children had positive rapid influenza assay results from April 21, 2009 to May 4, 2009. This knowledge led to the recommendation on May 4, 2009 that screening be performed only for patients with moderate to severe disease. With decreasing patient volumes, the decision was made to change MPERT staffing to 12 hours on May 7, 2009 and ultimately closed on that date, after seven operational days. Despite MPERT closing, additional physician coverage was provided in the ED for the next two days for any unanticipated surge in patient volume; because no such surge was observed, staffing returned to baseline. The MPERT was staffed by additional personnel working 12-hour shifts: one physician, three nurses (one of whom was responsible solely for laboratory follow-up and discharge education), one respiratory therapist, one to two patient care assistants, one laboratory technician and one housekeeper. A Spanish translator was always available because approximately 30% of the families cared for are Spanish speaking. One senior experienced nurse staffed the intake area. If needed, staff could be diverted to MPERT from other areas of the ED, depending on patient flow patterns. Staffing was accomplished through supplemental pay to physicians and nurses. Many nurses who worked in MPERT ordinarily worked in sections apart from emergency medicine. A hard-copy packet was created for streamlined documentation, including a nursing assessment sheet, preprinted physician order sheets, discharge instructions and school excuses. A new one-page history and physical documentation form and modified billing form were used. Charts were entered into the EMR within 24 hours of the patient visit. | | x | Hospital security staff and staff volunteers were trained to ensure compliance with cohorting efforts and also aided in escorting children and their families to their appropriate destinations. | |
| Burnette,  2009^[62]^ | 2 | | | x | | Trainees were informed of the requirement for completing the online pre-test using e-mail and through the online orientation. Trainees were made aware that the web-site tracked the viewing of lectures and that they were required to complete both the online post-test and a post-rotation questionnaire. | x | Before the development of this project, a literature review was undertaken to locate work done in the area of web-based medical education and, more specifically web-based education in the PED. Upon completion of the literature search, a needs assessment and an educational curriculum were created to define the goals and objectives of the project. | | | | | | |
| Gauthier,  2009^[63]^ | 2 | | | x | | The final guideline document was mailed to all physicians of the four divisions. | x | In 2003, a multidisciplinary team developed a CPG. The team included a general pediatrician; an ENT surgeon; an infectious disease specialist; a pediatric emergency physician; and a pharmacist. The CPG was developed by consensus within the group after reviewing the current medical literature. Before implementing the guideline, documents prepared by the CPG committee were submitted in writing (e-mail and paper) for comments to all members of the divisions involved in the care of children with refractory AOM, namely, emergency medicine, ENT, general pediatrics and infectious diseases. The guideline was also presented at division meetings by the committee’s chair. | | | | | | |
| Minniear,  2009^[64]^ | 2 | | | x | | Patients were informed that HIV screening was performed routinely for all adolescents and they were asked whether they would rather not be tested. Meetings with ED nursing administrators during the study period, to review the protocol for ease of use and interference with throughput time. | | | x | Visual computerized prompt was added to the electronic chart on September 8, 2008 to remind staff members to offer the screening to patients. | | | | |
| Kozer,  2009^[65]^ | 2 | | | x | | Physicians at Assaf Harofeh Medical Center were instructed to send blood for ethanol levels and urine for toxicological screening for any patient with one or more of the following symptoms or signs. Before the study commenced, physicians and nurses working in the ED were instructed regarding the study hypothesis and protocol. | | | x | Monthly reminders about the study hypothesis and the protocol were sent to all the senior physicians working in the ED. | | | | |
| Hayden,  2009^[66]^ | 2 | | | x | | Methods included small group interactive workshops. | | | x | Multiple visual prompts placed strategically in the department | | | | |
| Callegaro,  2009^[67]^ | 3 | | | x | | Posters explaining the guideline algorithm, a pocket guide with main recommendations and a care pathway were available for consultation. Implementation methods involved teaching sessions with doctors and nurses, mailing of the guideline to emergency staff and identification of a study coordinator at each site to provide further information. | x | The evidence-based guideline was selected after searching for guidelines, systematic reviews and meta-analyses in databases and libraries. The recommendations of the guideline were implemented independently in the two hospitals, taking account of the local context. Each hospital chose the educational strategies best suited to disseminate information about the guideline among staff members (posters and pocket guides at both centers and in addition ad hoc meetings in Padua and e-mails to attending doctors and residents in Paris). | x | To provide adherence support on a daily basis, the study coordinator attended the ED daily, reinforcing guideline principles and trying to resolve any problem inhibiting guideline implementation. | | | | |
| Morrissey,  2009^[68]^ | 1 | | | x | | A comprehensive staff education plan for nursing, physicians and pharmacy was developed and executed. Nursing education took place over several months and included a review of the CPG triage plan, assessment algorithm and templated orders. Education for ED physicians was provided during staff meetings by an attending hematologist, pain service physician or ED physician who had been involved in the CPG development. | | | | | | | | |
| Roukema,  2008^[69]^ | 1 | | | x | | All ED nurses received standardized training in how to use the CDSS. | | | | | | | | |
| Doherty,  2007^[70]^ | 3 | | | x | | Nursing staff were educated about the project during the afternoon handover each weekday in the week before the guideline was introduced. Education sessions were arranged to coincide with routine department teaching and updates about the project were provided informally at the end of other sessions. Information about the guidelines in the written orientation package and/or term descriptions that new and locum doctors received. | x | Identifying evidence practice gaps. Identifying barriers to change. | x | The use of reminders. Methods used included the guideline itself as a reminder in the notes, senior staff informally encouraging the use of the guidelines during clinical shifts, reminders during formal education sessions and notices placed on the walls and notice boards within the department. An implementation team, comprising the Director of the Department, the Nursing Unit Manager, a clinical nurse specialist, a clinical nurse consultant and a senior emergency physician, oversaw the project. | | | | |
| Boychuk,  2006^[71]^ | 1 | | | x | | During the last month of phase I, staff members at each participating ED were introduced to the protocols through 12 continuing medical education courses. Across multiple settings: Hawaii Child Asthma Research to Elevate Standards hosted 11 educational dinner discussions for 374 physicians, targeting those physicians whose patients had visited the ED multiple times during phase I, to promote asthma guideline awareness and compliance. | | | | | | | | |
| De Marco,  2005^[72]^ | 1 | | | x | | Three-hour training course designed by the authors that consisted in presentation of the CPGs, scientific evidence and rationale for CPG application and a discussion with case simulations. Two pediatricians acted as independent observers in the hospital ED. The independent observers trained in a two-step process: 1) they were interviewed to test their knowledge of the management of children with acute respiratory symptoms and 2) they were and trained in the methodology of recording data. | | | | | | | | |
| Buckmaster, 2005^[73]^ | 2 | | | x | | An education program was developed and targeted towards all doctors and nursing staff involved in the care of this patient group. This included all levels of medical and nursing staff, based in the EDs and the Children’s Ward. The program took the form of formal presentations over a three month period and the prominent display of posters in the relevant departments. Ongoing education in the form of posters and orientation to new pediatric residents and registrars continued throughout the final collection period. | x | Owing to difficulties in defining precisely when a CXR should be done, simple criteria were developed ‘a-priori’ to define when a CXR was unnecessary. The criteria were developed following review of the literature and discussion amongst pediatric staff. Discussions were held between the authors and registrars and consultants who worked in either Pediatrics or Emergency Medicine. The criteria developed were then reviewed and finally agreed upon by all of the five consultant general pediatricians who work in the area. | | | | | | |
| Buller-Close, 2003^[74]^ | 3 | | | x | | During the intervention phase II, physicians were asked to use the EDECS when treating any patients who presented with one of the specified chief complaints. During phase II, each physician on rotation in the ED was informed of the experiment, gave consent and was given a 15-min orientation solely regarding technical issues related to operating the software. | x | Before the study, essential items were identified, as those history and physical examination elements the value of which was required to navigate the process of care algorithms implicit in the clinical guidelines. | x | The quality of documentation of the medical record was characterized by measuring what percentage of charts contained each essential item. Before the study, we identified the essential items, those history and physical examination elements the value of which was required to navigate the process of care algorithms implicit in the clinical guidelines. Documentation on the aftercare instructions was similarly analyzed. | | | | |
| Lee,  2003^[75]^ | 2 | | | x | | Multidisciplinary team critically reviewed and modified existing protocol from another hospital, based on review of the literature and nationwide standards, guidelines and experience across disciplines. Compliance was maintained through education of those involved. Frequent in-services to the nursing staff, orientation guidelines to the surgical house officers and regular discussions with trauma nurse practitioners and emergency room personnel enhanced compliance of the pathway. | x | A short-term retrospective review (five months) was performed to assess initial performance. | | | | | | |
| Perlstein,  2002^[76]^ | 1 | | | x | | Dissemination of the guideline was accompanied by educational presentations to the medical staff (including medical grand rounds) and by a presentation at a monthly meeting held by community pediatricians, at house staff training sessions and in nursing training forums. The guideline was also mailed to all physicians on the hospital medical staff. Residents had access to original guideline references as needed. Formal education sessions were augmented with videotaped preservation of presentations for use in educational reinforcement sessions, poster displays summarizing the guideline highlights and summarization in hospital news publications. | | | | | | | | |
| Sharieff,  2001^[77]^ | 3 | | | X | | We implemented nursing in-services and departmental memoranda to stress the importance of early antibiotic administration in young febrile infants and to emphasize the goal of empiric therapy within two hour of patient presentation. | x | Review of hospital records to determine median time from triage to antibiotic administration. We set a goal of less than two hours from triage to antibiotic administration. | x | We then designed a Septic Infant Work-up Flow Sheet to serve as a checklist for procedures and allow the physician to immediately order the appropriate tests and antibiotics. To reinforce the 2 hour rule with the care team, the time by which antibiotics should be administered to the patient was written on the patient flow board. It became a team’s objective to meet or beat this time goal. | | | | |
| Gazarian,  2001^[78]^ | 3 | | | x | | Dissemination of guidelines to all medical managers, junior medical staff, nursing unit managers and pharmacists; use of opinion leaders to increase awareness and promote use of guidelines recommendations; targeted presentations delivered by peers (medical and nursing opinion leaders working in pairs) at regular interactive educational meetings. | x | Used a local consensus-building exercise to develop specific guidelines. Initial guidelines developed based on published research and applied to children with mild-moderate asthma. Insufficient evidence for severe acute asthma- sought input from a multidisciplinary team (e.g. ED staff, respiratory medicine, hospital management, etc.). This process ensured that the guideline content was acceptable to the majority and clear and easily understandable by all users. Endorsement of guidelines by the Clinical Services Committee of the hospital. | x | Reminders at the point of prescribing; audit and feedback of results to doctors and nurses in ED; important aspects of the handling of spacers were incorporated into the operating systems of individual wards so that they were routinely and reliably performed by ward assistants (supervision by trained nursing staff). | | | | |
| Schriger,  2000^[79]^ | 3 | | | x | | We created guidelines for various aspects of the management of febrile children and published baseline analyses and a clinical guideline for the care of children with fever without a source. Rules for other aspects of the care of pediatric patients were derived from standard text books. | | | x | Eligible patients were identified by the triage nurse who flagged the chart and attached a “Febrile child data collection form.” This form provided the physician with a list of the history and physical examination items that were required for all patients. The form was optional, was not part of the medical record and was included so that in the absence of bedside computing the physician would not have to go back to the bedside every time the computer asked a question for which the data had not been collected. After completing the history and physical examination, the physician would leave the bedside and, using the EDECS computer in the charting area of the ED, enter the history and physical examination results and order tests and treatments. | | x | Each physician on rotation in the ED was informed of the experiment, consented (all agreed) and was given a 15-min orientation, solely regarding technical issues related to operating the software. | |
| Lavelle,  1998^[80]^ | 1 | | |  | |  |  |  | x | The role of the medical staff was revised to influence the use of the trauma protocol. The primary role of seeing patient was shifted from Pediatric Emergency Physicians to trauma surgery group. | | | | |
| Rooholamini, 2017^[81]^ | 4 | | | x | | Pathway details were communicated through hospital-wide announcements and presentations at faculty meetings and teaching conferences. A web-based training module was required training for a subset of faculty in general pediatrics, hospital medicine and emergency medicine. Surgical and medical residents were encouraged (but not required) to complete the training. Pathway descriptions were included in nursing bulletins and reviewed at daily nursing huddles for two months after implementation. | x | Using standard processes and templates developed by our hospital’s Clinical Effectiveness team, we developed a clinical algorithm with an associated web-based training module. Process We formed a multidisciplinary team to design, implement and monitor the maintenance IVF pathway. This team was led by faculty from general pediatrics and hospital medicine and included physician and nursing representation from emergency medicine,  nephrology and general surgery. The team was supported by a centralized clinical effectiveness group, which included a medical librarian, informatician, consultant, project manager and data analyst. The standardized planning and implementation of our hospital’s clinical standard work pathways has been previously described. A systematic literature search from 2004 to 2014 was undertaken using the search terms and selection process referenced in our publicly available pathway. As evidence was synthesized, specific recommendations were developed and a pathway framework was created with phases for IVF initiation and monitoring. When evidence could not sufficiently address clinical questions, team members polled their respective colleagues and reported back to the group, with final decisions made using a Likert scale–based consensus tool. | x | A maintenance IVF PowerPlan was created and embedded into all PowerPlans that previously contained IVF orders. The PowerPlan can also be ordered on its own. | | x | Informaticians assisted with the development of a weight change calculator that displays on the “patient summary” page of the EMR and indicates both the absolute and relative (percentage) change between the last two consecutive measured weights. | |
| Hall,  2013^[82]^ | 2 | | | x | | Educational campaign (web-based presentation), which included a case study, explanation of consequences of contamination and teaching of new process for placing a sterile IV. | | | x | All contaminations reviewed on a biweekly basis and nursing staff were contacted via email regarding a contamination in which they were involved. | | | | |
| Zeretzke,  2012^[83]^ | 1 | | | x | | Participating physicians were trained to use the Florida State Health Online Tracking System immunization registry and completed an in-service to review the PED’s fever protocol used to operationally launch a fever without a source workup. | | | | | | | | |
| Volpe,  2012^[84]^ | 3 | | | x | | A team of process and subject matter experts was identified as champions for the change and was tasked with providing education to all personnel. A robust fever and neutropenia campaign was launched at a physician/nurse collaborative meeting in December 2008 where core knowledge and standards were communicated. Dedicated meetings with senior ED and oncology physicians and nurses led to engagement and reinforcement of the process. | x | The identification of key drivers was facilitated through a brainstorming session of ED physicians,  nurses and a pharmacist. | x | A group brainstorming session followed where clinicians were asked to identify possible improvements. Real time coaching and feedback were provided by champions and positive efforts were recognized and celebrated. Clinicians were informed of progress monthly via e-mail and visual postings on the unit. | | | | |
| Pakakasama, 2010^[85]^ | 2 | | | x | | All attending staff, fellows, residents, nurses and medical technologists involved were briefed about the guidelines in meetings. | x | The guideline committee was composed of staff from the Departments of Pediatrics, Emergency Medicine, Nursing and Pathology and the guideline was evidence-based. | | | | | | |
| Quint,  2009^[86]^ | 1 | | |  | |  |  |  | x | ED's electronic discharge system prompted the treating physician to refer eligible patients; (new care team): four full-time research assistants in the effort who also function as asthma educators. | | | | |
| Michalowski, 2004^[87]^ | 1 | | | x | | All users of the mobile emergency triage participated in short orientation sessions. | | | | | | | | |
| Muething,  2004^[88]^ | 4 | | | x | | The algorithm was shared at an ED divisional meeting and rolled out to the entire ED staff. Physicians on the development team, along with those already using the instrument, trained the rest of the staff. An ED nurse conducted similar educational sessions with nurses throughout the season. Weekly meetings with the improvement team were used to identify barriers and design solutions. | x | A multidisciplinary team was created and the emergency medicine physicians on the team led the development of point-of-care algorithms and rules specific to bronchiolitis care in the ED. | x | The algorithm was attached to the chart when a patient demonstrated symptoms of bronchiolitis. | | x | A respiratory scoring sheet and treatment recommendations were developed under the leadership of a respiratory therapist. Throughout the season, this leader met biweekly with therapists to increase communication, receive their input on what they thought was or was not working and to share the data. Respiratory therapists accompanied the physicians on morning rounds and were encouraged to make their recommendations verbally to the covering physician, based on the results of the respiratory assessment. | |
| Melzer-Lange, 2004^[89]^ | 1 | | |  | |  |  |  | x | The development and maintenance of a resource manual in close collaboration with the hematologists specializing in sickle cell disease was also an important part of assuring appropriate drug doses for each of our patients. | | | | |
| Dexheimer, 2014^[90]^ | 3 | | | x | | Physicians informed about the study in the operational emergency management, faculty and monthly resident meetings; an email from the ED director describing and supporting the study was sent out to the ED staff; respiratory therapists informed during their monthly management meetings; for a week prior to the study, the nurse leadership informed the nursing staff through the twice-daily meetings before the start of each shift. At all of these meetings, an investigator explained the study and answered any questions that arose. | x | A multidisciplinary respiratory distress committee was formed to develop an evidence-based practice guideline, including PED faculty and fellows, nursing staff, respiratory therapy, pharmacy and informatics personnel. | x | The computerized order set was an automatic prompt for intervention patients after the physician assigned an asthma score. | | | | |
| Jain,  2017^[91]^ | 4 | | | x | | The improvement team created an online resource document about JIL that was posted on the hospital internal web page and also shared in emails with the ED staff. This document included facts about JIL and IV-related pain, Web links to individually selected videos with instructions on using JIL and answers to common concerns identified on the pre-intervention survey. The improvement team recruited 10 experienced ED nurses and trained them to be JIL super users. With their assistance, we scheduled workshops for nurses and physicians to provide hands-on experience. | x | Before planning the intervention, we conducted an electronic, anonymous survey of ED physicians and nurses to assess their knowledge, attitudes and practices related to IV placement pain management in general and JIL use in particular. We planned our interventions based on expert opinion,  improvement team input, including the process map and ED personnel responses to the survey, including those depicted in the Pareto chart. We identified 6 opportunities for improvement and translated them into interventions, conducting multiple PDSA cycles and tests of change to modify and spread the interventions within the ED. | x | At baseline, the hospital had a standing order policy in place that allowed nurses to place specific orders (e.g., oral antipyretics, topical anesthetics for needlesticks) to improve efficiency. However, these standing order policies applied only until a medical provider evaluated the patient. This was a major barrier to JIL use because the need for most IV placements is determined after the initial medical evaluation. Providers often forgot to place the order and nurses found it burdensome to find and ask providers to request JIL orders. The standing order policy was modified to allow orders for topical anesthetics by the nurse even after the medical evaluation had taken place. Our last intervention was to place a small placard on all ED workstations to remind staff to consider ordering/using JIL with every IV. To emphasize the changes and provide positive feedback, we also sent reminder emails that included recognition for the nurse with the highest number of IVs placed with JIL. The option to order JIL was included in all order sets that had an IV placement option so that ordering JIL with each IV placement would be easier for the provider and, thus, occur more reliably. To emphasize the changes and provide positive feedback, we also sent reminder emails that included recognition for the nurse with the highest number of IVs placed with JIL. | | x | Technical assistance and facilitation for implementation processes: At our ED, JIL was initially stocked in only one of five zones, making access inconvenient for much of the ED and causing a barrier to wider use. Wall refrigerators were installed with assistance from the ED pharmacy and administration so JIL could be stocked in all ED zones. | |
| Fraser,  2018^[92]^ | 3 | | | x | | All participants received a take-home workbook and senior staff participated in a two-day interactive workshop. Short in-service presentations were also offered in participating departments. | x | A needs analysis was conducted with senior healthcare professionals at the study site prior to the development and implementation of the training. | | | | x | As a capacity-building program, senior staff who completed all components of the intervention were then qualified to implement ongoing training in the future, under the supervision of department directors. | |
|  |  | | |  | |  |  |  |  |  | | | | |
| Lee Gillespie, 2016^[93]^ | 3 | | | x | | The communication of the innovation was facilitated by the distribution of ink pens labeled on the ink pen barrel with “Workplace Violence: CHART IT TO STOP IT.” The diffusion process was spread over five months, allowing time for diffusion to occur. | x | Formal education to provide an overview of WPA, results from the pre-intervention survey, process for WPA reporting strategies to increase reporting and the rationale for reporting, was deployed during May 2015. | x | Charge nurse rounding to the five ED teams every four hours, reminding ED workers to report any incidents or near misses of any kind, including WPA using the ED’s safety event reporting “quick form” located throughout the ED. | | | | |
| Qazi,  2010^[94]^ | 1 | | |  | |  |  |  |  |  | | x | An asthma training program was administered to all of the emergency nurses preceding implementation of nurse initiated asthma care protocol over a period of three weeks. The program included PowerPoint presentations on pathophysiology, pharmacology and management of asthma that were presented by the principal investigator to groups of four to five nurses at a time. The management of asthma presentation included a description and demonstration of nebulization equipment. Respiratory therapy staff demonstrated salbutamol nebulization administration to the emergency nurses. The nurses were credentialed to administer the nebulizations by the respiratory therapy staff when they correctly performed all of the steps of nebulization administration on two patients. | |
| Hughes,  2013^[95]^ | 1 | | | x | | Training sessions were delivered to all ED staff (physicians, nurses, staff and security officers) to enhance positive interactions with patients and families, to emphasize the importance of follow-up outpatient care and highlight the seriousness of suicide attempts and suicide prevention. | | | | | | | | |
| Meunier-Sham, 2003^[96]^ | 3 | | | x | | Developing and distributing a printed matrix that outlined nonpharmacologic interventions; videos and posttests; education through group in-service sessions and reinforcement with posters and pocket cards. PainFree Pediatric boxes including distraction tools (e.g., bubbles, glitter wands and books) were provided to all clinical areas. | x | Needs assessment and in response the formation of a multidisc committee to develop appropriate strategies. Selection of appropriate EBIs such as pharmacological agents and nonpharmacologic agents. Adapting EBIs by responding to staff feedback. | x | Implemented a time-limited contest with gift certificates for nurses who continually used and documented “PainFree” interventions. Revisions to medical record systems: Protocol was supported by a preestablished order set in the computerized medical record. | | | | |
| Cunningham, 2009^[97]^ | 1 | | | x | | Research therapists were trained in motivational interviewing and skills training approaches at study onset, were monitored through monthly supervision and participated in retraining workshops throughout the study. | | | | | | | | |
| Einfeld,  2004^[98]^ | 3 | | | x | | In particular, YARDS focused on improving the availability of staff education and training, developing written policies and standard procedures for the clinical management of people with DSH and making cultural and attitudinal changes regarding DSH. | x | Staff from the project team worked closely with mental health service providers and EDs to develop and implement changes designed to enhance the service performance and clinical care for young people with DSH. | x | One feature of the YARDS project that may have contributed to its success was that YARDS adopted Total Quality Management as a tool for organizational change. This model focuses on the role of management in assisting employees to change their work practices and in ensuring the development of policies and procedures to support those changes. | | | | |
| Lemberg,  2005^[99]^ | 2 | | | x | | Pathway was implemented throughout the hospital as a management document along with an appropriate educational program. | x | Development of pathway by multidisciplinary team and trial period with feedback from stakeholders; pathway subsequently modified; "...grassroots engagement of all the stakeholders involved in the management of these children during the development phase of the CP was essential in physicians’ (both junior and senior) adhering to the pathway protocol. This approach was previously established as a key component in the success of CP adherence in our institution.” | | | | | | |
| Fox,  2008^[100]^ | 1 | | | x | | The program was provided as part of the regularly scheduled annual nursing education days in our health care organization. | | | | | | | | |

N: Number of; CT: Computerized tomography; ED: Emergency department; QI: Quality improvement; Min: Minutes; PICU: Pediatric intensive care unit; PED: Pediatric emergency department; TC: trauma center; RN: Registered nurse; PEM: Pediatric emergency medicine; CPG: Clinical practice guideline; VS: Vital sign; HCP: Health care professional; E-CDS: Electronic clinical decision support; CP: Clinical pathway; CDSS: Clinical decision support system; EMR: Electronic medical record; HSV: Herpes simplex virus; IV: Intravenous; EBI: Evidence-based initiative; CPETS: Chinese pediatric emergency triage system; F&I: Febrile immunocompromised; CBDI: Cancer and Blood Disease Institute; PDSA: Plan-do-study-act; VAT: Vascular access team; NIAP: Nurse-initiated analgesia pathway; PEM: Pediatric emergency medicine; HIV: Human immunodeficiency virus; ESI: Emergency severity index; MTP: Massive transfusion protocol; PCMC: Primary Children’s Medical Center; H1N1: Hemagglutinin type 1 and neuraminidase type 1; PAMG: Pediatric Acute Asthma Management Guidelines; MPERT: Mobile Pediatric Emergency Response Team; NET: Nose, ear and throat; AOM: Acute otitis media; CDSS: Clinical decision support system; CXR: Chest x-rays; EDECS: ED Expert Charting System; IVF: Intravenous fluids; JIL: Jet injection lidocaine; WPA: Workplace aggression; YARDS: Youth at risk of deliberate self-harm; DSH: Deliberate self-harm.
