# Supplementary material for: Implementation strategies in emergency management of children: A scoping review
Source: PLoS One. 2021 Mar 24;16(3):e0248826. doi: 10.1371/journal.pone.0248826 (PMC7990517; doi:10.1371/journal.pone.0248826)
Supplement: S4 Table — (DOCX) [file pone.0248826.s004.docx]

| **S4 Table. Effect of implementation strategies on the number of participants or effect estimates measured in the included studies** | | | | | | | |
| --- | --- | --- | --- | --- | --- | --- | --- |
|  | **Summary of implementation results** | | | | | **Conclusions** | |
| **First author, year of publication** | Direct (effects on healthcare providers measured outcomes) | | | Indirect (effects on patients measured outcomes) | | Direct | Indirect |
| McGrew, 2018^[8]^ | Guideline compliance was analyzed retrospectively and was generally well accepted by all providers, but because of the wide variability on patient injury patterns, no firm conclusions were made regarding compliance of individual providers. | | | Absolute reductions in head, chest and abdomen/pelvis CT scans were 17.7%, 11.5% and 18.8%, respectively, P<0.001. Percent positive head CTs were equivalent, but percent positive chest and abdomen CT increased after implementation. Secondary outcomes (hospital LOS, readmissions and mortality) were unchanged. | | Effective | Effective |
|  |  | | | | |  |  |
| McLaughlin, 2018^[9]^ | Survey response rate was 49% (93 of 190) pre-simulation, 22% (42 of 190) post-simulation and 79% (150 of 190) at two-year follow-up. These providers reported more anxiety, P=0.01 and less confidence, P=0.02 one-month post-simulation. At two-year follow-up, trained providers reported less anxiety, P=0.02 and greater confidence, P=0.01 compared to untrained providers. Implementation of an in situ multidisciplinary pediatric trauma simulation-based training program may initially lead to increased anxiety, but long-term exposure may lead to greater confidence. | | | | | Effective |  |
|  |  | | | | |  |  |
| Tavarez,  2017^[10]^ | | Admission rates by 19 physicians ranged from 15 to 30%, laboratory orders from 19 to 43%, pharmacy orders from 29 to 57% and radiology orders from 11 to 30%. There was no statistically significant difference in the proportion of patients admitted or with radiology or pharmacy orders placed between pre-intervention, P=0.58, intervention, P=0.19, or post-intervention, P=0.75 periods. There was a significant but very small decrease in laboratory orders between the pre-intervention and post-intervention periods. | | | | No significant benefit |  |
|  |  | | | | |  |  |
| Lee,  2018^[11]^ | The overall admission rate decreased from 58.2% (106 of 182) in the baseline period to 25.3% (65 of 257) after pathway revision, P<0.0001. | | | There was no significant difference in the percentage of patients returning to the ED within 72 hours and there were no adverse outcomes or deaths throughout the study period. During the 18 months after pathway revision, the median time to first epinephrine administration in the ED for Emergency Severity Index level I patients was consistently less than our target goal of less than 20 min. | | Effective | No significant benefit |
|  |  | | | | |  |  |
| Waddell,  2014^[12]^ | |  | | | There were a large number of children 52.5% (62) in the pre-test group vs. 47.9% (56) children in the post-test group who had no record of receiving any oral fluids during their ED care. | Effective | Negative effect |
| Johnson,  2018^[13]^ |  | | | Reductions were seen in ED LOS for treat-and-release patients from 3.9 to 3.3 hours, hospital LOS from 1.5 to 1.3 days, ED encounters requiring admission from 23.5 to 18.8%, admissions requiring ICU from 23.0 to 13.2% and total charges from $4457 to $3651. | |  | Effective |
|  |  | | | | |  |  |
| Puffenbarger, 2018 ^[20]^ | Overall CT use decreased from 37.7% (95%CI 31.7‒43.7) pre-implementation to 16.9% (95%CI 11.3‒22.5) post-implementation, P<0.001. Only 1% (95%CI 0‒2.9) of low risk patients received a head CT post-implementation compared to 22.6% (95%CI 16.1‒29.1) pre-implementation, P<0.001. CT use among patients ≥24 months decreased from 42.9% (95%CI 36.5‒49.6) to 19.6% (95%CI 13.1‒26.1), P<0.001 and remained low and unchanged for patients <24 months. Transfers to a pediatric trauma center and ED returns within 72 hours were unchanged, while median ED LOS improved from 1.5 to 1.3 hours, P=0.03. There were no missed clinically important traumatic brain injury after implementation of the guideline. | | | | | Effective |  |
| Lukes,  2019^[21]^ | By the end of the implementation period mean time to antibiotic improved to 53 min and patients received antibiotics within 60 min 83% of the time. The primary aim of decreasing time of triage to antibiotic administration showed a significant difference between the baseline cohort 0 and three other cohorts, χ2(4)=75.1, P<0.01,with a cohort 0 median of 108 min, P<0.01, cohort 1 median of 77 min, P=0.039, cohort 2 median of 91 min, P=0.891 and cohort 3 median of 47 min, P<0.01 with the sustainability cohort four having a median of 51 min, P<0.01. | | | | | Effective |  |
|  |  | | |  | |  |  |
|  |  | | | | |  |  |
| Carson,  2018^[22]^ | There were significant increases in provider knowledge and confidence scores for child physical abuse screening and recognition, P<0.001. The educational session and Escape Instrument were the most reported screening facilitators and transition to a new electronic health system was the most reported barrier. Of the 14 ED HCPs who completed the Project Evaluation Survey, 78.6% (11 of 14) supported system-wide implementation of the ED child physical abuse screening program. | | | There were no child physical abuse diagnostic codes entered by ED HCPs in the 30-day period before implementation of the screening program, which was similar to the same 30-day period in the preceding year. In the 30-day period following project implementation, there were three child physical abuse diagnostic codes entered by ED HCPs, compared with two in the same 30-day period the preceding year. Because of the small sample sizes, no statistical analysis was conducted. | | Effective | No significant benefit |
| Libetta,  1999 ^[23]^ | | In the study group, 56.76% (432 of 761) patients received radiography compared with 63.93% (500 of 782) in the control group. There was a statistically significant reduction in radiography rate of 7.2% (95%CI 2.3‒12.1, P<0.01). The sensitivity of the Ottawa ankle rules was 98.3% and the specificity 46.90%. There was no increase in the number of missed fractures. | | | | Effective |  |
| Hendrickson, 2018^[24]^ | | Among community ED patients with imaging, US use increased from 36 to 51%, P=0.049 and CT scan use decreased from 81 to 66%, P=0.044 in the post-intervention period. No change in complications or safety outcomes. | | | | Effective |  |
| Norton,  2007^[25]^ | | The overall rate of hospital admission decreased from 27.5% (10 of 74) in the pre-implementation group to 13.5% (53 of 193) in the post-implementation group, number needed to treat=7.1, P=0.02. All reduction in hospitalization occurred in children with moderate to severe asthma exacerbation. After implementation of the CP, the rate of administration of oral corticosteroids to patients with moderate or severe exacerbations increased from 71 to 92%, P=0.01 and significantly more patients received b2-agonists in the first hour, P=0.02. | No significant change in relapse to acute care occurred within two weeks, P=0.19. | | | Effective | No significant benefit |
| Dona, ^[26]^  2018 | | In outpatients, post-CP broad-spectrum antibiotic regimens (mainly macrolides) decreased from 50 to 26.8%, P=0.02. Narrow-spectrum (amoxicilin) increased. Decrease of median DOT from 10 to 8 days, P<0.0001 and median LOT from 10 to 8, P<0.0001. No difference in treatment failure was found from 2.3 to 11.8%, P=0.29. In inpatients, post-CP: Broad-spectrum regimens decreased from 100 to 66.7%, P=0.02; introduction of narrow-spectrum regimens occurred from 0 to 33%, P=0.02; the antibiotic courses decreased in hospitalized patients, median DOT from 18.5 to 10 days, P=0.004; no statistical difference was found in LOT, median LOT from 11 to 10 days, P=0.06; lower days of broad spectrum therapy, median broad-spectrum DOT from 17 to 4.5 days, P<0.0001; no difference in treatment failure was found - changed from 16.7 to 15.4%, P=1. Physicians prescribed narrow-spectrum monotherapy more frequently than broad-spectrum combination therapy, DOT/LOT ratio=1.157 pre-CP vs. 1.065 post-CP. | | | | Effective |  |
| Mohan,  2018^[27]^ | | There was a statistically significant reduction in rates of CXR ordering after pathway implementation and ED LOS but no change in other diagnostic testing or cardiology consultation. Follow-up in our health care system for pediatric chest pain increased from 15 to 29% with implementation, but none of these visits resulted in the diagnosis of a new cardiac condition. There were no instances identified where use of the pathway resulted in missed cardiac disease. | | | | Effective |  |
| Jones,  2017^[28]^ | | Shorter stays in ED resulted in no change in the time to cranial CT scan, no reduction in the proportion of appropriate scans and no change in the high proportion of abnormal findings on CT. | | | | No significant benefit |  |
| Murray,  2017^[29]^ | | The mean time to urine collection for all infants in this study was 150 min. Comparing pre-implementation to post-implementation, the mean time to urine collection was reduced by 23 min from 160 to 137 min (95%CI -35.5‒-10.9). The mean time to lumbar puncture for all infants in this study was 239 min. However, there was no statistically significant reduction in mean time to lumbar puncture when comparing pre to post-implementation data with a difference of 8 min from 243 to 235 min (95%CI -8‒24.2). The mean time of arrival to the first antibiotic administration for all infants in this study was 270 min. The mean time to the first antibiotic administration was reduced by 36 min (95%CI -58.2‒-13.3). When infants younger than 29 days were analyzed separately, the mean time to the first antibiotic was reduced by approximately 50 min (95%CI -86.7‒-15.1). Because antibiotics were not recommended by the pathway for all older infants (29–56 days), only those who received antibiotics were included in the analysis and the mean time to the first antibiotic administration was reduced by approximately 30 min (95%CI -58.6‒-1.29). | | | | Effective |  |
| Geurts,  2017^[30]^ | | Implementation of the clinical decision support system proved a high compliance rate. The standardized use of oral rehydration solution significantly increased from 52 to 65% (RR=2.2, 95%CI 1.09‒4.31, P<0.05). | We observed no differences in other outcome measures (e.g. costs, LOS at ED). | | | Effective | No significant benefit |
| Ahmad,  2017^[31]^ | | We found a significant decrease in ED testing for HSV among patients who did not meet guideline criteria, P<0.01. After guideline implementation, there was no increase in the proportion of patients receiving HSV testing when indicated by the guideline. No change was observed in the use of HSV blood or cerebrospinal fluid polymerase chain reaction, or initiation of acyclovir in the ED in patients meeting the guideline criteria for testing. | | | | No significant benefit |  |
| Gildenhuys, 2009^[32]^ | | There was an increase in spacer use from 17 to 26% (95%CI 2‒16, P=0.015) and a reduction in ipratropium use from 58 to 44% (95%CI 22‒5, P=0.0029). The proportion of patients treated with corticosteroids did not change. The number of patients with an ED action plan increased. The number of CXRs ordered decreased and the hospital admission rate decreased. Prior to the introduction of the asthma CPGs worksheet, the use of the asthma guidelines was not documented. After the implementation, it was documented in 50.7% (145 of 286) medical records that the guidelines had been used. | | | | Effective |  |
| Rutman,  2016^[33]^ | | Provider adherence to the asthma pathway was high (79–88%). | There was clinically and statistically significant decrease in ED LOS for admitted patients by ∼30 min, P=0.006 and there was a nominal (<10%) increase in costs of asthma care for patients in the ED, P=0.04. | | | Effective | Effective |
| Lin,  2016^[34]^ | | The Chinese pediatric emergency triage system experienced by the experimental group was associated with a reduced patient flow through the pediatric emergency room, Cox‑Stuart Test, t=0, P<0.05, a higher triage rate from 90.75 to 93.40%, χ2 = 801.546, P<0.001, better triage accuracy from 85.09 to 96.32%, χ2 = 710.904, P<0.001, shorter overall wait times from 41.60 ± 15.40 to 37.30 ± 13.80 min, t=11.27, P<0.001, markedly shorter wait times for severe patients from 3.23, IQR=1.90‒4.36 to 2.07, IQR=0.65‒4.11 min, z=-2.057, P=0.040. | Higher family satisfaction rates from 92.21 to 94.23%, χ2 = 321.528, P<0.001. | | | Effective | Effective |
| Shah,  2016^[35]^ | | The negative appendectomy rate was 5.6% in the pre-implementation cohort compared with 8.7% in the post-implementation cohort, P=0.09. The mean time the patient spent in the ED before implementation was 6.2 hours compared with 5.8 hours after implementation, P=0.06. The rate of CT utilization was cut by two-thirds from 75.4 to 24.2%, P<0.0001. Utilization of US before CT increased from 24.4 to 95.3%, P<0.0001. Surgical consultation before CT increased from 14.7 to 76.1%, P<0.0001. The frequency of operative intervention without any imaging increased from 1.8 to 9.9%, P<0.0001. | | | | Effective |  |
| Dandoy,  2016^[36]^ | | At baseline, only 25% of patients received antibiotics within 90 min and the median time to antibiotics was 135 min. On October 31 2011, the definition of successful timely delivery of antibiotics was transitioned from 90 min to 60 min. The median time to antibiotics remained relatively stable at 40 min from January 2012 to September 2014 (sustainability period). In 2014, we noted the majority of our failures were close to goal in the ED. | | | | Effective |  |
| Cohen,  2016^[37]^ | | Pre-protocol TTA was 96.9 ± 57.8 min and 35% of patients received antibiotics within 60 min. After implementation of the protocol TTA decreased to 64.3 ± 28.4 min, P<0.0001 with 51.4% receiving antibiotics within 60 min. | | | | Effective |  |
| Fallon,  2015^[38]^ | | Appy-Score use increased from 24% (37 of 155) in July to 89% (226 of 254) in September, P<0.001. Appendicitis incidence in the pre-implementation group was 23.1%. Of all US exams performed for suspected appendicitis in the post-implementation period, 299 of 985 were reported without using the structured reporting template or scoring system. Appendicitis incidence in these 299 patients was 33.4% (100 of 299) vs. 41.7% (286 of 686) in the post-implementation study population, P=0.015. The use of the Appy-Score system did not significantly change the diagnostic performance of US exams between the pre- and post-implementation groups with the exception of the negative predictive value, which decreased from 98.8% (95%CI 97.7‒99.3) pre-implementation to 96.7% (95%CI 94. ‒-98.1) post-implementation, P=0.012. | | | | No significant benefit |  |
| Jeong,  2015^[39]^ | | Team to address febrile oncology/BMT patients upon arrival. This intervention increased our median percentage of patients receiving timely antibiotics to its highest rate (95%). | After implementation, the rate of return visits was reduced significantly, from 4.4 to 2.6%, P<0.01. The admission rate for return visits was also reduced, but not significantly so, from 22.3 to 17.5%, P=0.37. | | |  | Effective |
| Dexheimer, 2014^[40]^ | | The Bayesian network identified 1339 patients with asthma exacerbations, of which 788 had an asthma diagnosis determined by an ED physician-established reference standard (positive predictive value=69.9%). The median time to disposition decision did not differ among the intervention, 228 min, IQR=141‒326 and control group, 223 min, IQR=129‒316, P=0.362. The hospital admission rate was unchanged between intervention (25%) and control groups (26%), P=0.867. | The ED LOS did not differ among intervention, 262 min, IQR=165‒410 and control group, 247 min, IQR=163‒379, P=0.818. | | | No significant benefit | No significant benefit |
| Higginbotham, 2014^[41]^ | | Implementation of the algorithm was related to a significant increase in algorithm required screenings: skeletal survey, P<0.001, urinalysis, P<0.001 and transaminase levels, P<0.001. Children with government-subsidized or no insurance were more likely to be screened for child abuse via skeletal survey before the algorithm implementation compared with those with private insurance, OR=2.7 (95%CI 1.2‒6.0, P=0.017). This relationship did not exist after the algorithm implementation, OR=1.2 (95%CI 0.56‒2.46, P=0.66). Final determination of child abuse was related to insurance status both before and after the algorithm implementation. The guideline recommended the use of fundoscopic eye examination to screen for retinal hemorrhage only in those children with positive findings for intracranial hemorrhage, what caused decrease of fundoscopy eye exam from 35.2 to 8.1% and increased the rate of positive retinal hemorrhage findings from 6.8% of all screened before the implementation to 15% of those screened following the implementation. | | | | Effective |  |
| Geurts,  2014^[42]^ | | Before implementation, compliance to the diagnostic strategy according to local protocols was correct in 24.9% (42) patients compared with 46.7% (70) after implementation of the guideline, P<0.001. In the post-intervention group, dipstick tests were significantly more used, 28.4 vs 50.7%, P<0.001. | | | | Effective |  |
| Boutis,  2013^[43]^ | | The physician followed the rule recommendations for 81.4% (350) of these patients. The reported reasons for non-adherence in the remaining 80 were as follows: fear of missing a significant fracture, 31.3% (25), physician preferred the Ottawa Ankle Rules, 13.8% (11), family wanted a radiograph, 10.0% (8), patient was difficult to examine, 7.5% (6) and no reason documented, 37.5% (30). No significant differences in physician satisfaction. | | | | Effective |  |
| Taylor,  2013^[44]^ | | At post-intervention, significantly more patients received nurse-initiated analgesia from 3.0 to 43.9%, P<0.001 and the median time to analgesia was significantly reduced from 58 to 23 min, P<0.01. Also, significantly more patients received ‘adequate analgesia’ post-intervention from 41.2 to 72.5%, P<0.001. At follow up, the proportion of parents who were very satisfied with their child’s overall pain management trended upwards in the post-intervention period from 47.1 to 66.7%, P=0.07. | | | | Effective |  |
| Russell,  2013^[45]^ | | Among patients who had appendectomy in the year before implementation 90%(of 70) had CT scans, 6.9% (of 70) had US and 5.7% (of 70) had no imaging. The negative appendectomy rate before implementation was 5.7%. In patients undergoing appendectomy in the post-implementation cohort, 48% (of 96) underwent CT, 39.6% (of 96) underwent US and 15.6% (of 96) had no imaging. The negative appendectomy rate was 5.2%. We demonstrated a 41% decrease in CT use for patients undergoing appendectomy at our institution without an increase in the negative appendectomy rate or missed appendectomy. | | | | Effective |  |
| Hack,  2013^[46]^ | | Seventy-five percent (224) patients accepted HIV testing in the first three months post-implementation. Review of HIV testing performed in the PED from October through December 2008 showed 39 patients aged 13 to 20 years were tested. Routine testing increased the number of patients tested by 446%. | | | | Effective |  |
| Wolff,  2012^[47]^ | | Median time to phototherapy, historical control: 128 min vs. postintervention group: 52 min, P<0.001; median time to bilirubin result, 157 vs. 99 min, P<0.001; and median ED LOS, 268 vs. 195 min, P<0.001 were shorter for neonates treated after the implementation of the CP. | | | | Effective |  |
| Doyle,  2012^[48]^ | | After the intervention, patients experienced a significant decrease in arrival-to-triage times compared with the pre-intervention group, P<0.001 with most patients, 88.3%, being triaged in less than 10 min after the intervention. Following implementation of fast track guidelines, patients were 14% more likely to be triaged to fast track compared with pre-intervention patients, OR=1.14 (95%CI 1.11‒1.67). Additionally, patients with the lowest acuity were nearly 50% more likely to be triaged to fast track compared with post-intervention patients, OR=1.47 (95%CI 1.35‒1.63). | | | | Effective |  |
| Waseem,  2012^[49]^ | |  | Overall ED visits increased by 93.6% (95%CI 78.2‒109.6, P<0.001). Fifty-two patients tested positive for H1N1. The walkout rate was 2.9% (95%CI 1.9‒4.0) in 2009 compared with the walkout rate of 1.5% (95%CI 1.0‒2.0) in 2008. There were no statistically significant differences between walkouts, P=0.06 and seven-day asthma revisits, P=0.07 in 2008 and 2009 despite the almost doubling of the ED visits. Admission rates from 2009 did not significantly differ from 2008 from 11.2% (990 of 8842) vs. 10.2% (464 of 4560), P=0.07. | | |  | No significant benefit |
| Hendrickson, 2012^[50]^ | |  | Seventy-two percent of MTP patients had at least one coagulation value outside of the normal range upon ED arrival and the median time to FFP transfusion decreased fourfold after MTP implementation, P<0.0001. A total of 49% of MTP patients received greater than 70 mL/kg blood products and the 24-hour median FFP-RBC transfusion ratio was twofold higher in these patients than the pre-MTP cohort, median 1:1.8 vs. 1:3.6, P=0.002. No improvement in mortality was observed after MTP implementation, taking into consideration injury severity, prothrombin time and partial thromboplastin time. | | |  | No significant benefit |
| Crocker,  2012^[51]^ | |  | Patient-recalled pain scores of the ED visit in the protocol group were significantly lower than those of the pre-protocol group, Wong-Baker Faces Pain Scale 5.07 vs. 4.01, P<0.001 yet parent estimates of pain did not show a significant change at any point. Patient assessment of pain at ED discharge did not show a significant change either, Wong-Baker Faces Pain Scale 1.99 vs. 1.56, P=0.09. | | |  | Effective |
| Angoulvant, 2012^[52]^ | | Antibiotic use related to ARTI decreased significantly, P<10^-4^, after implementation of the 2005 guidelines. Overall, the mean relative decrease was 35%. Antibiotic prescriptions decreased in 75% (6 of 8) of the ARTI categories, but did not decrease for pneumonia or AOM. Antibiotic prescriptions for ARTI/1000 visits decreased significantly, P<0.0001 from 51.4 in 2005/2006 to 40 in 2008/2009. The proportion of antibiotic prescriptions for tonsillitis was significantly higher in children aged three years or older, as age is a criterion for antibiotic prescription, 46 vs. 34%, Chi Square Test, P<0.0001. The antibiotic most often prescribed for ARTI during the study period was amoxicillin/clavulanic acid, but the percentage of amoxicillin/clavulanic acid prescriptions in children with pneumonia fell from 36 to 29% between 2005/2006 and 2008/2009, P<0.0001. In children with AOM, the proportion of cefpodoxime proxetil prescriptions increased from 15 to 20% between 2005/2006 and 2008/2009, P=0.0005. | | | | Effective |  |
| Larsen,  2011^[53]^ | | One notable improvement occurred in obtaining a complete set of vital signs from the first encounter, which improved from 83 to 98%, P=0.05. Antibiotic administration within three hours improved from 53 to 81% and serum lactate determination which improve from 10 to 81%. | The median hospital LOS declined over the study period from 181 to 140 hours, P<0.05. There was no change in mortality rate, which averaged 6.3% (22 of 345). | | | Effective | Effective |
| Cruz,  2011^[54]^ | | When compared with children seen before the protocol, time from triage to first bolus decreased from a median of 56 to 22 min, P<0.001 and triage to first antibiotics decreased from a median of 130 to 38 min, P<0.001. | | | | Effective |  |
| Iyer,  2011^[55]^ | | After implementation of this process, 95% of the patients with long-bone extremity fractures treated with IV opioids received a first dose within 45 min of arrival, compared with a pre-intervention baseline average of 20%. | | | | Effective |  |
| Fagbuyi,  2011^[56]^ | |  | Overall ED daily volumes increased by a mean of 51.8% (113). The mean wait time decreased from 92.9 to 81.2 min (95%CI 10.2‒13.2). Overall mean ED LOS decreased from 241 to 212.3 min (95%CI 25.8‒31.6). Rates of elopement were unchanged. Rates of return were unchanged within 48 hours, 3.0% in 2009 vs. 2.9% in 2008, OR=1.03 (95%CI 0.91‒1.18) and within seven days, 6.2% in 2009 vs. 5.7% in 2008, OR=1.09 (95%CI 0.99‒1.20). The use of the rapid screening process required a mean of 23.5 (95%CI 16.4‒30.6) additional hours per day of physician staffing and a mean of 26.3 (95%CI 18.5‒34.1) additional hours of nursing staffing. | | |  | Effective |
| Fein,  2010^[57]^ | | During implementation, identification of adolescents with psychiatric problems increased significantly, 4.2 vs. 2.5%, OR=1.70 (95%CI 1.38‒2.10), as did ED assessments by a social worker or psychiatrist, 2.5 vs. 1.7%, OR=1.47 (95%CI 1.13‒1.90). Of the 857 patients who were screened with the behavioral health screening-ED 10.5% (90 of 857) were identified as having psychiatric problems, OR= 4.58 (95%CI 3.5‒-5.94) and 8.3% (71 of 857) were assessed, OR=5.1 (95%CI 3.80‒6.88). | | | | Effective |  |
| Babl,  2010^[58]^ | | Nitrous oxide and ketamine were the most commonly used agents. Midazolam use decreased over the study. Hospitals relevant proxy markers of sedation safety were significantly improved over the pre-implementation level. Three years after implementation markers of sedation safety were still improved over pre-implementation levels. However, based on a minimum compliance with seven of 11 sedation safety markers both sites deteriorated, Royal Children’s Hospital from 96 to 80%, P=0.028 and Sunshine Hospital from 68 to 32%, P=0.001. | | | | Effective |  |
| To,  2010^[59]^ | | Compared to the retrospective group, patients in the prospective group received an average of 0.23 (95%CI 0.03‒0.44, P=0.0283) more doses of salbutamol in the first hour during the ED stay. Their odds of receiving oral corticosteroids, AOR z=2.26 (95%CI‒1.63-3.14, P=0.0001) and SaO_2_ reassessment before ED discharge, AOR z=2.02 (95%CI 1.45‒2.82, P=0.0001) were also twice as high compared to the retrospective group. | | | | Effective |  |
| Trottier,  2010^[60^ | | Treatment used in the ED changed over the 10-year period. More patients were treated in the ED in 2006–2007. In addition, there were more patients who received intravenous medications (mainly prochlorperazine) in 2006–2007. Fewer patients were discharged home without a prescription for medication in 2006–2007 compared with 1996–1997. The medications prescribed were also different between the two periods. Principally, more NSAID were used in 2006–2007 compared with 1996–1997. | | | | Effective |  |
| Cruz,  2010^[61]^ | | All MPERT patients were discharged home. The percentage of patients who left without being seen decreased by 44%, from 143 to 81 patients with the opening of MPERT and only <1% (3) of MPERT patients left without being seen, P<0.001. 4.5% (16) children of MPERT volume were mistriaged and had to return to another section of the ED for additional evaluation; reasons included nine of children needing additional laboratory evaluation, five requiring IVF and two requiring breathing treatments. Of these 16, three stayed overnight in the observation unit (two with dehydration; one with wheezing) and one child with pneumonia and hypoxemia was admitted to the inpatient unit. | | | | Effective |  |
| Burnette,  2009^[62]^ | | The mean change in pre- and post-test score was a 1% improvement for trainees who viewed no lectures and a 6.2% improvement for those who viewed lectures (95%CI 2.5‒7.9). In the linear regression model, the estimate of the coefficient was 0.43, P<0.001, meaning that for each lecture viewed, the post-test score rose by 0.43%. 111 residents and fourth year medical students participated in the program. An initial 32 completed testing before implementation of the on-line lectures (March 2007‒August 2007) and another five did not complete the on-line lectures after implementation (September 2007‒February 2008). Seventy-one completed testing and on-line lectures and all but three completed at least 10 on-line lectures during their rotation. 14 of 111 trainees did not complete the pre- or post-test (including two who viewed the lectures). Fourty-two (30 of 72) of the trainees who completed the online lectures and testing returned surveys. All of them were comfortable using the Internet and 87% (26 of 30) found the site easy to use. Ninety-three percent (28 of 30) felt that the program would be helpful on in-training or board examinations. All felt that their educational goals were met and that the format would be useful in other areas of education. | | | | Effective |  |
| Gauthier,  2009^[63]^ | | After the implementation of the guideline, the children were significantly more likely to have received an adequate dose (mg/kg) of ceftriaxone, COR=5.6 (95%CI 2.5‒12.5), but the proportions of children receiving ceftriaxone for an adequate indication, COR=1.4 (95%CI 0.6‒3.5) and receiving an adequate number of doses of ceftriaxone, COR=0.9 (95%CI 0.4‒1.8) were not significantly different. Overall, after the implementation of the guideline, the patients were twice as likely to receive adequate treatment with ceftriaxone, COR=2.2 (95%CI 0.5‒9.0); this finding was not, however, statistically significant. | | | | Effective |  |
| Minniear,  2009^[64]^ | | Screening rates were consistently low during the five-month period before initiation of computerized prompting, that is, 28.9% (1056) of patients compared with 54.3% (946) after initiation, P<0.0001. The HIV screening acceptance rate was 86.7% (95%CI 85.1‒88.1) among all patients approached and 89.5% (95%CI 88.1‒90.9) with the exclusion of patients who reported previous HIV testing. | | | | Effective |  |
| Kozer,  2009^[65]^ | | A total of 47% (27) of patients in the younger age group and 37% (56) in the older age group tested positive for ethanol, P=0.15. 10% (6) of patients in the younger age group and 8% (12) patients in the older age had a positive urine drug screen, P=0.55. Introducing structured guidelines for ordering toxicological screening increases the detection of alcohol and drug of abuse among adolescents presenting to PEDs. | | | | Effective |  |
| Hayden,  2009^[66]^ | | There was no statistically significant difference in the PTTT between observation periods. The PTTT was, on average, 16% (95%CI 17‒33, P=0.302) longer after the implementation of the features of serious illness in children checklist. | | | | No significant benefit |  |
| Callegaro,  2009^[67]^ | | The hospitalization rate significantly decreased respectively, from 57.3 to 20.5% and from 16.9 to 3.2%, without any concomitant increase in readmission rate. Readmission cases were never due to severe bacterial infections. The proportion of patients who received blood examinations decreased significantly | | | | Effective |  |
| Morrissey,  2009^[68]^ | | There were statistically significant improvements in use of 4 of the 5 endpoints measured in the CPG, i.e. pain scale from 57 to 99%, P<0.001, appropriate analgesic dose by weight from 53 to 78%, P<0.001, utilization of patient-controlled analgesia (PCA) from 71 to 87%, P<0.01, and time, median (min) to initiation of PCA from 393 (10th, 90th percentile, 210, 820) to 245 (135, 540), P<0.001. | | | | Effective |  |
| Roukema,  2008^[69]^ | | Compliance with registration of febrile children was 49% (683 of 1399). Adherence to the advice to order laboratory tests was 82% (61 of 74). | | | | Effective |  |
| Doherty,  2007^[70^ | | At 12-month follow-up, there were significant increases in the documentation of asthma severity from 45 to 90%, P<0.001, use of spirometry from 32 to 66%, P=0.012, use of spacers from 5 to 53%, P<0.001 and use of written short-term asthma management plans from 16 to 69%, P<0.001. There was a reduction in the use of ipratropium bromide in mild asthma from 31 to 3%, P<0.001. There was no significant change in the use of systemic steroids from 74 to 62%, P=0.29, or antibiotic use in afebrile patients from 15 to 6%, P=0.175. For the seven clinical indicators combined, compliance with the guideline increased from 47 to 79%, P<0.001. | | | | Effective |  |
| Boychuk,  2006^[71]^ | | From pre-intervention to post-intervention, the number of patients possessing a written asthma action plan increased from 48 to 322. Of 186 persistent asthmatics, 34 were using controller medications daily, 34 as needed and 118 not at all. Daily use increased to 80 three weeks post-intervention and to 68 three months post-intervention. | | | | Effective |  |
| De Marco,  2005^[72]^ | | In phase I, 26.2% were admitted to the hospital. In phase II, 16% of the children with influenza-like symptoms were admitted by CPG-trained pediatricians and 25.8% by control pediatricians. Training of ED pediatricians in the application of a specific CPG may result in a substantial decrease of the admission rate. In fact, the number of inappropriate admissions by CPG-trained pediatricians was almost half that by control pediatricians. | | | | Effective |  |
| Buckmaster, 2005^[73]^ | | Twelve months prior to the education program, 466 children presented with asthma: 55.8% (260 of 466) had a CXR, of which 81.1% (211 of 260) were unnecessary. During the six month period following implementation of the program 197 presented with asthma: 36.5% (72 of 197) had a CXR, of which 78% (56 of 72) were deemed unnecessary. However the percentage of all children presenting with asthma who had an unnecessary CXR fell from 45.3% (211 of 466) to 28.4% (56 of 197), P=0.00005. There was also a decrease in the admission rate from 46% before to 31% after the period of education. | | | | Effective |  |
| Buller-Close, 2003^[74]^ | | Before participation, the majority of the 198 physicians who completed the survey believed that current practice was in “near-complete” or “total” agreement with existing guidelines for occupational exposure to blood and body fluid=72%, fever in children=62% and low back pain=52%. Most believed that the care they provided was “very good” or “excellent” for occupational exposure to blood and body fluids=70%, fever in children=75% and low back pain=64%. The post-participation questionnaire was completed by 86% (78 of 91) of eligible physicians who used the ED Expert Charting System 2 or more times. For all three modules, physicians uniformly agreed that aftercare instructions produced by the ED Expert Charting System were superior to standard handwritten instructions for occupational exposure to blood and body fluids=100%, fever in children=97% and low back pain=97%. There was heterogeneity of belief across complaints about all other aspects of the ED Expert Charting System. Seventy-two percent of surveyed physicians believed that the ED Expert Charting System was “better” or “much better” than standard care for occupational exposure to blood and body fluids; 46% held this belief for low back pain and 35% for fever in children. | | | | Effective |  |
| Lee,  2003^[75]^ | | After implementation of the pathway, time required for cervical clearance in non-intubated children decreased from before, 12.3 ± 1.5 vs. after, 7.5 ± 0.9 hours, P=0.014. A clear trend toward earlier clearance in intubated patients existed from before (n= 6), 40.0 ± 16.8 vs. after (n=6), 19.4 ± 8.1 hours, P=0.10. Neither group had missed injuries. | | | | Effective |  |
| Perlstein,  2002^[76]^ | | The mean total encounters for acute gastroenteritis decreased by 23% after the guideline was implemented, from 2072 to 1603, P<0.07. The percentage of admitted children with minor illness decreased significantly. | Mean LOS decreased significantly for children with minor illness. Mean hospital costs did not change significantly | | | Effective | Effective |
| Sharieff,  2001^[77]^ | | The July 1998 review revealed an approximate 25% reduction in the time interval from presentation at triage to antibiotic administration. The implementation of our practice protocol resulted in the following improved median time intervals: triage to room, 15 min (95%CI 10‒20, P=0.12); room to antibiotic administration, 90 min (95%CI 80‒100, P=0.008); and triage to antibiotic time, 105 min (95%CI 95‒115, P=0.006). | | | | Effective |  |
| Gazarian,  2001^[78]^ | | Physician adherence to guideline recommendations was 94.4% in the first month, 90.3% in the second month and 100% in the third month of the post-implementation period. 94.4% (151) were initially treated with a spacer device in 1999. In 1997, no children were initially treated with spacers in the ED. Documented reasons for using a nebuliser rather than spacer in eligible children in the ED were severity of asthma, child's inability to use a spacer, parent preference and physician preference. | | | | Effective |  |
| Schriger,  2000^[79]^ | | Mean percentage documentation of 21 essential history and physical examination items increased from 80% during the baseline period to 92% in the intervention phase (95%CI 10‒15). Mean percentage documentation of 10 items in the after-care instructions increased from 48% at baseline to 81% during the intervention phase (95%CI 28‒38). All documentation decreased to baseline when the computer system was removed. | | | | Effective |  |
| Lavelle,  1998^[80]^ | | Protocol implementation resulted in an increase use of investigation, e.g. CT scan, OR=8.4 (95%CI 2.3‒30), number of subspecialty consultation from three to 20%, P<0.02 and time spent in the ED from three to three hours, which has little effect on patient care and increased cost of care. | | | | Negative effect |  |
| Rooholamini, 2017^[81]^ | | The use of isotonic maintenance IVF increased significantly from 9.3 to 50.6% (95%CI 39.8‒42.8), whereas the use of any hypotonic fluid decreased significantly from 94.2 to 56.6% (95%CI 239.0‒236.2). Compared with the pre-implementation period, during the post-implementation period there were significant increases in the number of potassium-containing bags administered in the ED from 52.9 to 75.3% (95%CI 19.4‒25.6) and the proportion of patients who had serum sodium monitoring within 36 hours of IVF initiation and a daily weight measurement. Chart reviews of pathway-eligible patients with severe dysnatremia in the pre- and post-implementation periods revealed an increase in IVF-associated dysnatremia from two to four cases, respectively. | | | | Effective |  |
| Hall,  2013^[82]^ | | The peripheral blood culture contamination rate was reduced from 3.9% during the baseline period to 1.6% during the intervention period, P<0.0001 with yearly estimated savings of $250 000 in hospital charges. | | | | Effective |  |
| Zeretzke,  2012^[83^ | | Registry access reduced the percentage of screening blood draws from 100 (91) to 42% (95%CI 37‒53, P<0.001). | | | | Effective |  |
| Volpe,  2012^[84]^ | | Mean time to antibiotic delivery in febrile oncology patients with known neutropenic status dropped from 99 min in the pre-implementation period to 49 min in the post-implementation period, whereas it dropped from 90 to 81 min in possibly neutropenic patients. The percentage of patients who met the 60 min target for time to antibiotics rose from 50 to 88.5%. | | | | Effective |  |
| Pakakasama, 2010^[85]^ | | Secondly, adverse outcomes were compared (intervention was effective). |  | | | Effective |  |
| Quint,  2009^[86]^ | |  | Over the six-month follow up period, patients in the intervention group had significantly fewer ED visits than did patients in the control group, 0.64 vs 1.19, ARR=0.54 (95%CI 0.40‒0.72). Patients in the intervention group also had significantly fewer total unscheduled visits to any source (ED or elsewhere), 1.39 vs. 2.34, ARR=0.60 (95%CI 0.46‒0.77). The intervention group showed significantly more symptom-free periods, decreased asthma severity at one-month follow-up and improvements in several measures of quality of life that largely persisted over the six-month follow-up period. | | |  | Effective |
| Michalowski, 2004^[87]^ | | Accuracy of the system in triaging patients was assessed. The overall accuracy of the system was found to be comparable to those of experienced physicians. | | | | Effective |  |
| Muething,  2004^[88]^ | | Comparison of use of bronchodilator therapy (intervention was effective), guideline order sets (intervention was ineffective), resource utilization (intervention was effective). | LOS (intervention made no difference) and readmission (intervention made no difference). | | | Effective | No significant benefit |
| Melzer-Lange, 2004^[89]^ | | Patients treated using the protocol had initiation of PCA therapy within 35 ± 7 min from the last bolus narcotic dose in the ED vs. 211 ± 17 min for non-protocol patients. The mean number of narcotic bolus doses and the mean length of hospital admission were not significantly different between the two groups. ED-PCA administration was preferred by 92% (23 of 25) over inpatient initiation of PCA. | | | | Effective |  |
| Dexheimer, 2014^[90]^ | | Hospital admission rate (intervention made no difference). Time to disposition (intervention made no difference). | ED LOS (intervention made no difference). | | | No significant benefit | No significant benefit |
| Jain,  2017^[91]^ | | Before the initiative, only 11% of IV placements were associated with JIL use. Within 14 measurement cycles (seven months) of the first intervention, 54% of IV placements were associated with JIL use. During the monitoring period after the final intervention (24 cycles, 12 months), the proportion of IV placements with JIL remained ≥50%. We observed an increase in nurse initiated JIL orders from 15% during the baseline period to 60% of all JIL orders after the interventions. The number of JIL devices ordered by providers also increased during this period. | | | | Effective |  |
| Fraser,  2018^[92]^ | | Detection and clinical response (intervention was effective). |  | | | Effective |  |
| Lee Gillespie, 2016^[93]^ | | Overall reporting decreased from 53 to 47%, P=0.06. Reasons for reporting were severity of incident and being asked to report. Reasons for not reporting were incidents being too minor and no action would be taken. WPA reporting significantly decreased worsened for threats of aggression committed by patients from 75 to 39.3, P<0.01.WPA reporting significantly increased (improved) for assaults committed by patients from 55.9 to 90.9, P=0.02. ED workers were most likely to notify other employees in non-administrative roles of the aggression they experienced. When WPA was a threat of aggression by a patient, ED workers were most likely to report the WPA to security/police. | | | | No significant benefit |  |
| Qazi,  2010^[94]^ | | The mean door-to-first-salbutamol-nebulization-time was 59.8 ± 38.8 min for the pre-group and 28.5 ± 26.6 min for the post-group (95%CI 23.0‒39.6). A reduction of 22.8 min in the mean door-to-steroids-time also was observed (95%CI 8.8‒36.9). The door-to-second-nebulization and door-to-bedside-nurse time intervals were reduced by 21.7 min (95%CI 9.1‒34.4) and 15.6 min (95%CI 7.5‒23.7), respectively. No medication errors were reported in either group. | | | | Effective |  |
| Hughes,  2013^[95]^ | | The Family Intervention for Suicide Prevention was shown to significantly increase the likelihood of youths receiving outpatient treatment, including psychotherapy and combined medication and psychotherapy. | | | | Effective |  |
| Meunier-Sham, 2003^[96]^ | | The initial low rate of documentation of PainFree interventions, 16%, has increased to 70%, P<0.001. These results have been sustained over a six-month period despite the lack of further initiative programs such as gift certificate awards. Actual use of PainFree options versus documentation of PainFree options appears closer to 90%. | | | | Effective |  |
| Cunningham, 2009^[97]^ | | Of those screened, 26% (637) of adolescents screened positive. Ninety-seven percent of those randomized to a brief intervention self-reported that they found one intervention section “very helpful.” At posttest, significant reductions in positive attitudes for alcohol use and violence and significant increases in self-efficacy related to alcohol/violence were found for both therapist and computer interventions. Readiness to change was not significantly improved. | | | | Effective |  |
| Einfeld,  2004^[98]^ | |  | The service improvements made during the YARDS project were maintained four years after the project ended, which was from a mean of 209.3, SD=46.7 to 270, SD=22.0. At the four-year follow-up, the mean Service Activity Scale score for services that participated in the YARDS project was 270.17, which was greater than the mean for services that did not participate in the YARDS project of 240.13. An independent samples t-test revealed that this difference was significant, t(11)=2.257, P<0.05. | | |  | Effective |
| Lemberg,  2005^[99]^ | | Full blood count was more likely to be performed prior to the introduction of the pathway, 77.1%, than after pathway introduction, 66.8%, P<0.004. Urine microscopy and culture also was significantly decreased from 56.3 to 40.4%, P<0.0005. Median patient costs were reduced from $1228 to $752 following pathway introduction, P<0.0001; however, rates of admission were increased from 18.6 to 28.8%, P<0.0001. LOS decreased but was not statistically significant. | | | | Effective |  |
| Fox,  2008^[100]^ | | The aggregate mean pretest score was 71%; the mean posttest score was 91% and two-year posttest score was 67%. Thus, two years after the presentation, overall knowledge about pediatric disaster had decreased among the group. | | | | No significant benefit |  |

CT: Computerized tomography; CI: Confidence interval; ED: Emergency department: LOS: Length of stay: Min: Minute; HCP: Health care provider; US: Ultrasound; CP: Clinical pathway; DOT: Days of therapy; LOT: Length of therapy; CXR: Chest x-ray; RR: Relative risk; HSV: Herpes simplex virus; CPG: Clinical practice guideline; IQR: Interquartile range; TTA: Time to admission; OR: Odds ratio; HIV: Human immunodeficiency virus; PED: Pediatric ED; H1N1: Hemagglutinin type 1 and neuraminidase type 1; MTP: Massive transfusion protocol; FFP: Fresh-frozen plasma; RBC: Red blood cell; ARTI: Acute respiratory tract infections; AOM: Acute otitis media; IV: Intravenous; AOR: Adjusted OR; SaO_2_: oxygen saturation; NSAID: Nonsteroidal anti-inflammatory drugs; MPERT: Mobile Pediatric Emergency Response Team; IVF: Intravenous fluids; COR: Crude OR; PTTT: Presentation-to-treatment time; PCA: Patient-controlled analgesia; ARR: Adjusted RR; JIL: Jet injection lidocaine; WPA: Workplace aggression; YARDS: Youth at risk of deliberate self-harm; SD: Standard deviation.
