# Supplementary figures and images for: Implementation strategies in emergency management of children: A scoping review
Source: PLoS One. 2021 Mar 24;16(3):e0248826. doi: 10.1371/journal.pone.0248826 (PMC7990517; doi:10.1371/journal.pone.0248826)

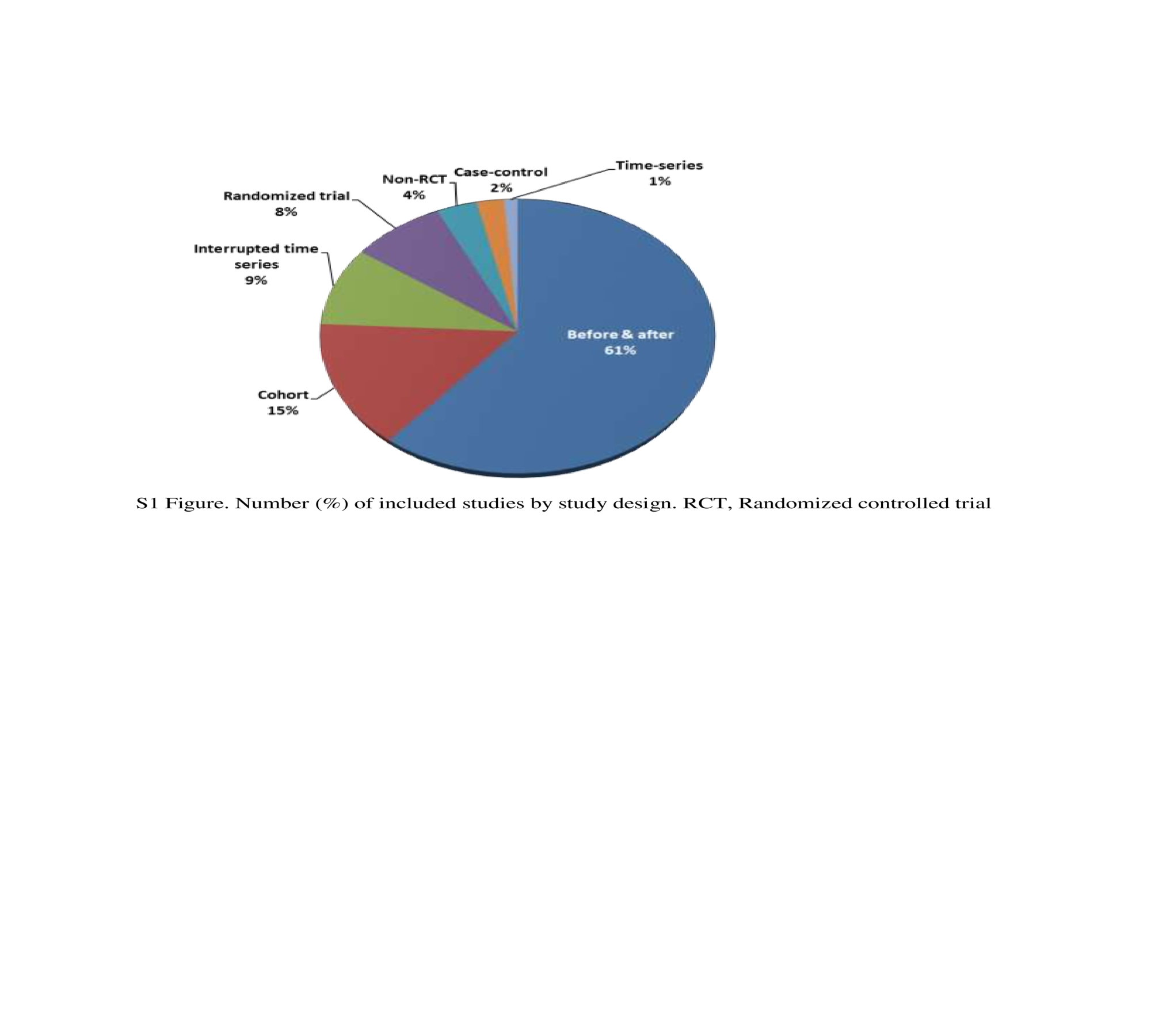

Supplement: S1 Fig — RCT, Randomized controlled trial. (TIFF) [file pone.0248826.s005.tiff]
